# IFN-β production promotes metabolic rewiring and protection against oxidative stress in hepatitis delta virus-infected hepatocyte cultures

**DOI:** 10.1038/s41419-025-07838-z

**Published:** 2025-07-18

**Authors:** Olga A. Khomich, Patrick Giavalisco, Romain Parent, George S. Krasnov, Peter Tessarz, Philip Meuleman, Rani Burm, Natalia F. Zakirova, Jennifer Molle, Enkhtuul Batbold, Eyal Gottlieb, Fabien Zoulim, Alexander V. Ivanov, Birke Bartosch

**Affiliations:** 1 Lyon Hepatology Institute; INSERM Unit 1350 PaThLiv; Université Claude Bernard Lyon 1, Lyon, France; 2 Engelhardt Institute of Molecular Biology, Russian Academy of Sciences, Moscow, Russia; 3 Max Planck Institute for Biology of Ageing, Cologne, Germany; 4 Department of Human Biology, Radboud Institute for Molecular Life Sciences, Faculty of Science, Radboud University, Nijmegen, The Netherlands; 5 Laboratory of Liver Infectious Diseases, Department of Diagnostic Sciences, Faculty of Medicine and Health Sciences, Ghent University, Ghent, Belgium; 6 Department of Cancer Biology, University of Texas MD Anderson Cancer Center, Houston, Texas USA; 7 Hepatology Department, Hospices Civils de Lyon, Lyon, France

**Keywords:** Infection, Viral hepatitis

## Abstract

Type I interferons are secreted in response to various stimuli and are used as a treatment for many diseases, including infections with the hepatitis B virus (HBV) and its satellite virus, hepatitis delta (HDV). HDV significantly aggravates HBV-mediated liver damage and is – in contrast to HBV - a strong inducer of interferon responses, including IFN-β. As the role of IFN- β in liver metabolism is so far ill explored, we studied its impact on hepatocyte metabolism in HDV-infected cultures. Transcriptome analysis, isotope tracing and functional tests on differentiated, HDV-infected hepatocytes showed reduction of mitochondrial TCA cycle and respiratory activity and increases in serine, asparagine and glutathione synthesis. Furthermore, the stress-response factor ATF4 was activated by IFN-β via yet unidentified non-canonical mechanisms and mediated resistance to oxidants. IFN-β furthermore reduced the expression and activity of liver differentiation markers. Thus, IFN-β-mediated dedifferentiation and stress-resistance may contribute to HDV-associated liver pathology.

## Introduction

The liver regulates metabolite interconversion and storage, energy transformation and detoxification. Despite the fact that interferons type I (IFN-α, IFN-β, etc) and III (IFN-λ1-4) are secreted in response to a wide spectrum of stimuli and used as treatment for pathologies including chronic infections with hepatitis B and hepatitis D viruses (HBV, HDV), their effects on liver metabolism remain ill defined. It has previously been shown that IFN-β treatment of primary mouse hepatocytes reduced transcript levels of genes involved in glutathione (GSH), amino acid, drug, fatty acid and cofactor metabolism, while upregulating expression of tryptophan 2,3-dioxygenase [1]. Several reports suggest that IFN-α decreases hepatic cytochrome expression and activity [2, 3]. In mice infected with lymphocytic choriomeningitis virus, the expression of a ROS-scavenging enzyme, superoxide dismutase, 1 in the liver was found to be downregulated in an IFN type I-dependent manner [4]. Moreover, treatment with IFN-α caused liver damage in mice even in the absence of infection, suggesting that activation of the type I IFN pathway per se may be harmful for the liver. Overall, the impact of IFN type I and III on liver metabolism remains poorly understood.

Chronic hepatitis D (CHD) is known to be associated with strong IFN-β and IFN-λ production [5]. HDV is a defective hepatotropic RNA virus that can replicate by itself but requires hepatitis B virus (HBV) surface antigens for envelopment and spread. HDV therefore utilises the same route of infection as HBV, involving binding to hepatocyte receptor sodium-taurocholate cotransporting polypeptide (NTCP) [6]. Thus, HDV-chronic carriers are always co-infected with HBV. There are an estimated 15-20 million HBV/HDV-coinfected people worldwide, with the exact number unknown due to the absence of a common screening practice [7]. A frequent outcome of chronic HBV monoinfection is the development of fibrosis, cirrhosis and hepatocellular carcinoma (HCC) [8, 9]. Chronic HDV/HBV infection is significantly more aggressive than HBV monoinfection and marked by accelerated disease progression, but the underlying mechanisms remain unclear. In approximately 70% of cases, chronic HDV/HBV leads to cirrhosis development within 5-10 years. HDV increases the risk of cirrhosis threefold [10] and the incidence of HCC up to 9 fold compared to HBV monoinfection [11, 12]. One key to HDV-aggravated liver pathology could be the pronounced immune responses and, in particular, melanoma differentiation-associated protein 5 (MDA5)-mediated IFN-β and IFN-λ production [13], which are known to be triggered by HDV, but not HBV [14]. Furthermore, IFN therapy, which is still in use for treatment of CHD as monotherapy or in combination with emerging antivirals [15], is known to be accompanied by general/systemic adverse effects such as flu-like symptoms, fatigue, neuropsychiatric symptoms, elevated transaminase levels and others [16]. Despite the use of IFN as a therapeutic agent and its potential role in CHD, it remains unclear how IFNs affect hepatocytes and liver functionality.

Here, we used in vitro cultures infected with HDV and studied the effect of virus-induced IFN on hepatocyte metabolism and physiology. Amongst the available in vitro models, differentiated liver progenitor HepaRG cells best mimic primary human hepatocytes (PHH) in terms of quiescence, functional innate immunity, detoxification and mitochondrial metabolism (high mitochondrial versus low glycolytic activity) and offer the advantage of strong data reproducibility in contrast to PHH [17]. Furthermore, as the HBV receptor NTCP is expressed in HepaRG only upon 4 weeks of differentiation, HepaRG ectopically expressing NTCP were used for infection with HDV in most experiments to shorten the process.

Characterization of the main metabolic pathways in HDV-infected differentiated HepaRG^NTCP^ cultures revealed IFN-dependent reduction of mitochondrial functionality. Furthermore, metabolic stress response-related pathways were activated downstream of IFN-β. The observed metabolic adaptations resulted in reduced hepatocyte functionality and resistance to oxidants, features that may play key roles in the accelerated liver pathology in CHD and give important clues regarding the effects of IFN-β on liver physiology.

## Results

### HDV-induced IFN activates the metabolism of amino acids and glutathione, but downregulates pathways feeding the TCA cycle

**Fig. 1:**
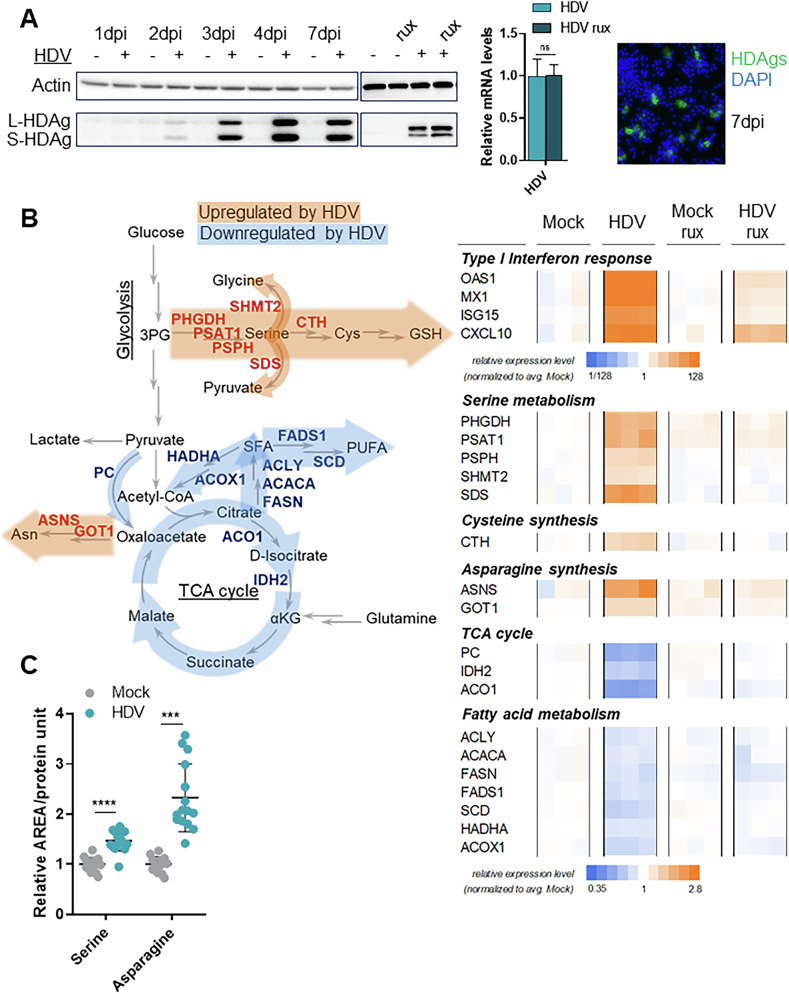
The major metabolic pathways impacted by HDV infection. **A** Left panel: Representative immunoblot analysis of mock and HDV-infected HepaRG^NTCP^ cells treated or not with 1 μM ruxolitinib (rux) for the last 4 days of infection and analysed at the indicated dpi (7 dpi for rux treatment). Middle panel: Relative HDV RNA levels in HepaRG^NTCP^ cells treated or not with 1 μM rux for the last 4 days of infection at 7dpi. Right panel: HDV-positive cells were visualized via immunofluorescence by HDAgs (green) and nuclear DAPI (blue) staining at 7dpi. *n* = 2. **B** A simplified metabolic scheme with HDV-affected pathways; an RNAseq-based heatmap depicting relative expression levels of selected genes normalized to average mock condition. Saturated fatty acids (SFA); polyunsaturated fatty acids (PUFA). **C** Serine, asparagine and reduced gluthatione (GSH) quantification in mock and HDV-infected HepaRG^NTCP^ cells. *n* = 3. Statistical analysis: for **C** data are shown as mean +/- SD. For serine and GSH t-test and for asparagine Mann-Whitney test were used. ****p < 0.0001; ***p < 0.001.

To analyse the impact of IFN signalling on liver metabolism, differentiated HepaRG or HepaRG^NTCP^ cells were infected with HDV, treated with the interferon signalling inhibitor ruxolitinib (rux) or not and harvested at 6-7 days post infection (dpi), when HDV antigen expression was at maximal levels (Fig. 1A). HDV RNA and protein levels were not sensitive to treatment with rux, which blocks JAK1 and JAK2 signalling **(**Fig. 1A**)**. As few data in the literature are available on metabolic changes induced by interferon and HDV, a global RNAseq analysis was performed in the presence or absence of rux (Fig. 1B**;** Fig. S1A). Approximately 3000 genes were differentially regulated by HDV. Infection upregulated type I IFN signalling, as previously reported [18], an effect that was reversed by rux treatment (Fig. 1B). Serine, cysteine and asparagine synthesis were induced, while expression of genes involved in the TCA cycle and fatty acid (FA) metabolism was downregulated (Fig. 1B). Major pathways such as glycolysis, gluconeogenesis and purine metabolism were not affected. An upregulation of de novo serine biosynthesis genes (*PHGDH*, *PSAT1* and *PSPH*), as well as serine dehydratase (*SDS*) responsible for serine catabolism into pyruvate, and serine hydroxymethyltransferase 2 (*SHMT2*), which links serine metabolism to the folate cycle was detected. However, KEGG pathway analysis showed a slight downregulation of transcripts of genes involved in folate metabolism. Serine can also be utilized for cysteine synthesis *via* the transsulfuration pathway, consistent with the observed increase in cystathionine gamma-lyase (*CTH*) transcripts. Synthesis of asparagine was activated, indicated by upregulation of asparagine synthetase (*ASNS*) and glutamic-oxaloacetic transaminase 1 (*GOT1*). In line with the transcriptome data, the levels of serine and asparagine, as well as GSH, for which cysteine is a precursor, were higher in HDV-infected cultures (Fig. 1C). Transcript levels of aconitase (*ACO1*) and isocitrate dehydrogenase 2 (*IDH2*) responsible for α-ketoglutarate (αKG) formation in the TCA cycle, were lower in HDV-infected cells (Fig. 1B). The carbon backbone used for TCA cycle metabolites originates from three main sources: glutamine, pyruvate, and fatty acids. Pyruvate can enter the TCA cycle either directly *via* pyruvate carboxylase (PC)-mediated conversion to oxaloacetate, or be used for acetyl-CoA synthesis by pyruvate dehydrogenase (PDH). *PC* expression was decreased in HDV-infected cells. PDH activity is regulated by pyruvate dehydrogenase kinase (PDK); no changes in PDH phosphorylation were observed (Fig. S1B). Acetyl-CoA is also the endpoint metabolite of FA oxidation (FAO). Transcriptome analysis revealed downregulated expression of genes involved in saturated and polyunsaturated FA (SFA and PUFA, respectively) synthesis as well as degradation (Fig. 1B). All these data were verified by RT-qPCR and western blotting in additional infections using HepaRG^NTCP^ as well as fully differentiated HepaRG cells (Fig. S1C, D). Consistent with the fact that HDV is not sensitive to rux treatment, various metabolic inhibitors did not affect HDV replication (Fig. S1E).

**Fig. 2:**
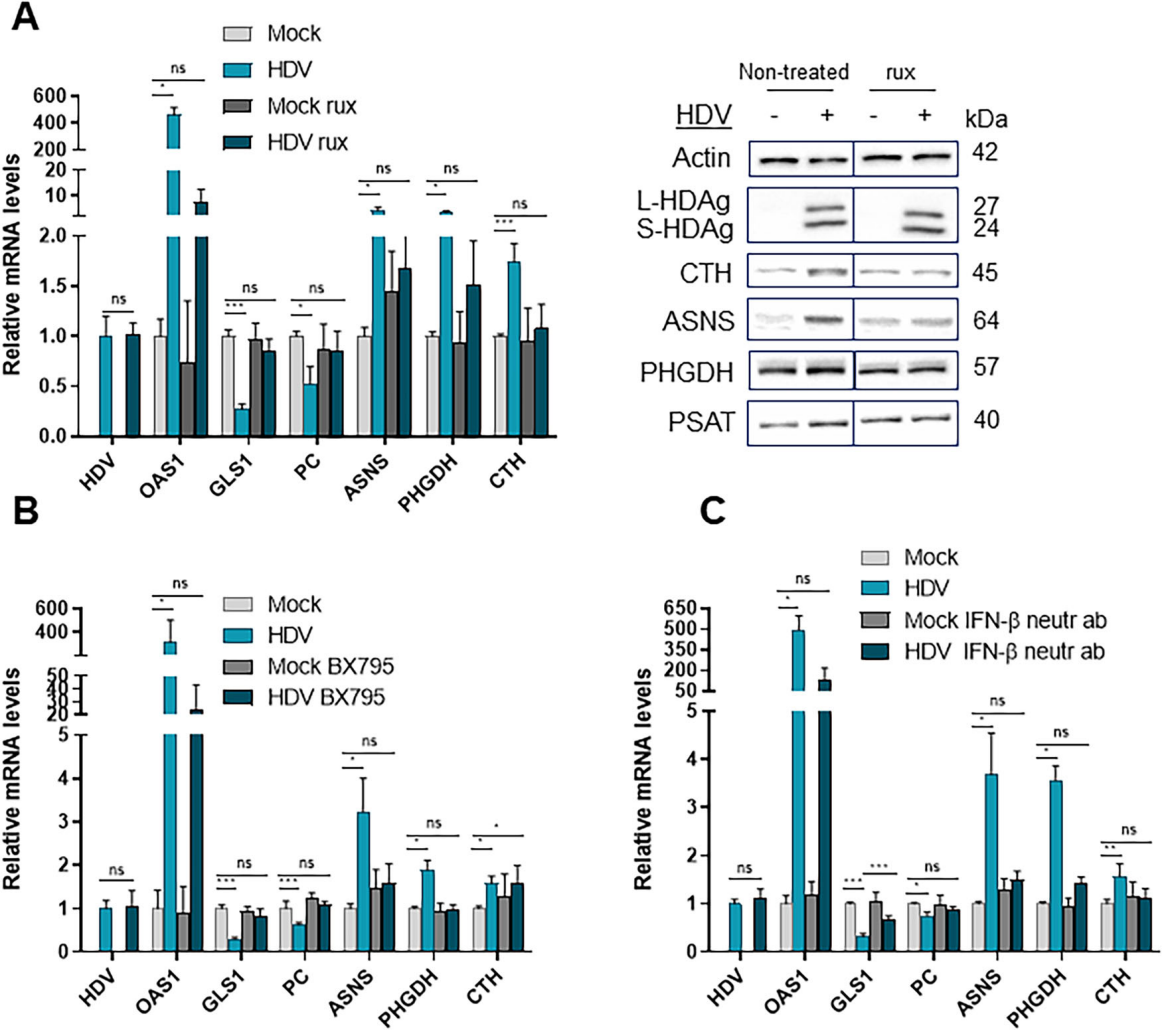
HDV-induced metabolic changes are reversed by inhibiting IFN-β signalling. **A** Relative mRNA (left panel) and protein (right panel) levels in HepaRG^NTCP^ cells treated or not with 1 μM rux for the last 4 days of infection. Normalized to non-treated mock cells, *n* = 3. The non-treated panel of the immunoblot is also used in Fig. S1C and Fig. 4D. **B** Relative mRNA levels in HepaRG^NTCP^ cells treated or not with 3 μM BX795 for the last 4 days of infection. Normalized to non-treated mock cells, *n* = 3. **C** Relative mRNA levels in HepaRG^NTCP^ cells treated with 1 μg/mL IFN-β-neutralizing antibodies starting 1 dpi. Normalized to non-treated mock cells, *n* = 3. Statistical analysis: data are shown as mean +/- SD. t-test (for HDV levels) or one-way ANOVA or one-way ANOVA on ranks with Tukey multiple comparison test were used. ****p < 0.0001; ***p < 0.001; **p < 0.01; *p < 0.05.

**Fig. 3:**
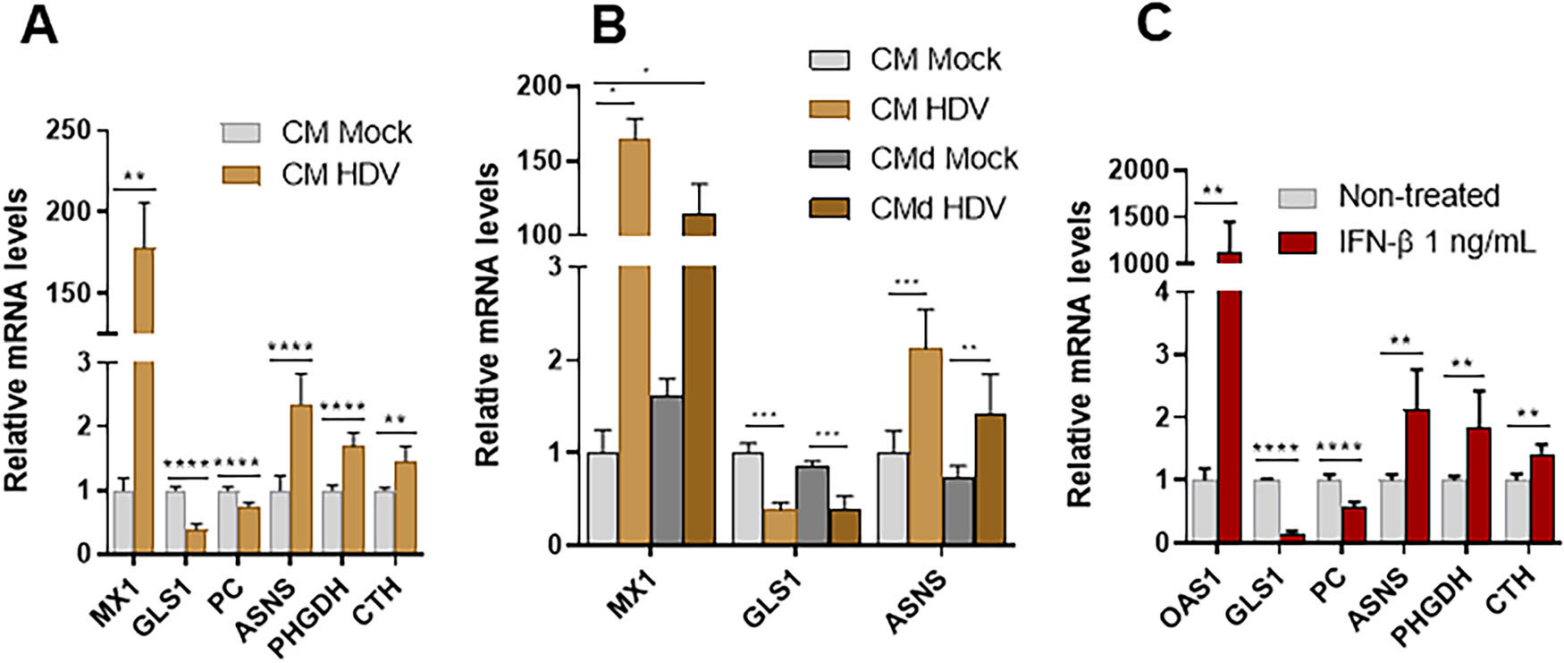
HDV-induced metabolic changes are mediated by IFN-β. **A** Relative mRNA levels in HepaRG^NTCP^ cells treated with conditioned medium (CM) for 3 days. Normalized to CM mock cells, n = 3. **B** Relative mRNA levels in HepaRG^NTCP^ cells treated with conditioned medium, dialyzed (CMd) or not (CM), for 3 days. Normalized to CM mock cells, *n* = 3. **C** Relative mRNA levels in HepaRG^NTCP^ cells treated or not with 1 ng/mL of IFN-β for 3 days. Normalized to non-treated cells, n = 3. Statistical analysis: data are shown as mean +/− SD. t-test or Mann-Whitney test were used. ****p < 0.0001; ***p < 0.001; **p < 0.01; *p < 0.05.

The role of IFN in HDV-induced metabolic changes was furthermore validated with BX795, which blocks IFN signalling via TBK1 **(**Fig. 2A, B**)**, as well as neutralizing antibodies to IFN (Fig. 2C). Anti-IFN-β reversed the metabolic phenotype of infected cells for most targets, suggesting that IFN-β is secreted and is responsible for most, if not all, metabolic changes that were induced by HDV. Treatment with conditioned cell medium harvested from HDV-infected cells at 7 dpi and dialysed or not (2-kDa cut-off) or with recombinant IFN-β induced very similar metabolic changes, confirming that IFN-β is the main mediator of metabolic host cell responses to HDV in vitro (Fig. 3A–C). Incubation with recombinant IFN-α or IFN-λ showed similar effects, pointing to a shared metabolic phenotype induced by type I and III IFNs in hepatocytes (data not shown). Taken together, these results suggest that HDV-induced IFN-β promotes asparagine, serine and cysteine/GSH synthesis and modifies TCA cycle activity.

**Fig. 4:**
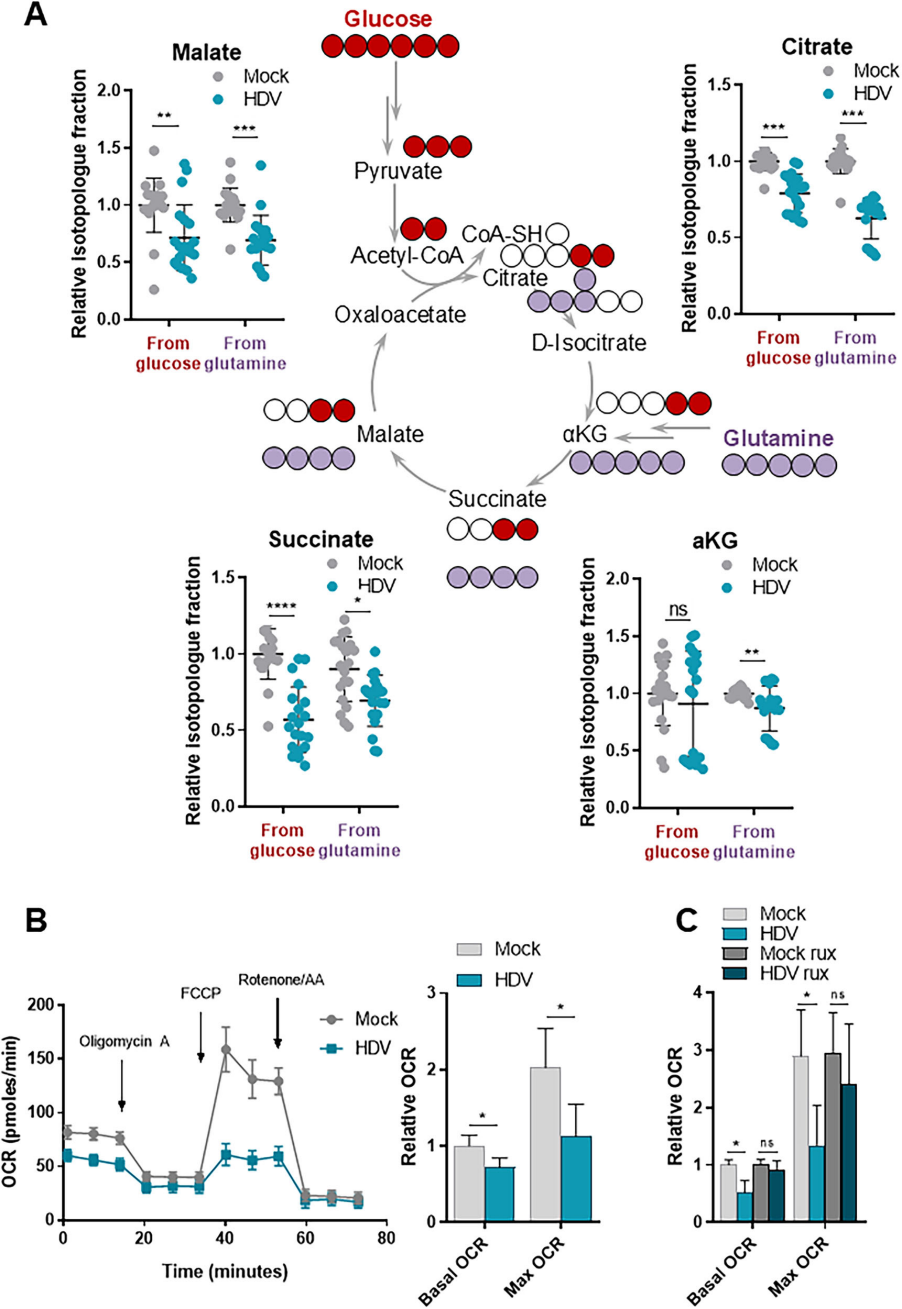
HDV infection inhibits the TCA cycle activity and respiration. **A** Isotope tracing. Scheme: distribution of ^13^C carbons from tracers (^13^C_6_ glucose, red; ^13^C_5_ glutamine, violet) in TCA cycle metabolites. Graphs: relative isotopologue fractions measured in mock and HDV-infected HepaRG^NTCP^ cells treated with glucose-^13^C_6_ or glutamine-^13^C_5_ for the last 10 h or 3 h of infection, respectively. Isotopologues used for the measurement from glucose/glutamine: citrate M + 2/M + 4; αKG M + 2/M + 4; succinate M + 2/M + 4; malate M + 2/M + 4. Mean +/− SD, normalized to mock cells, *n* = 4. **B** Oxygen consumption rates (OCR) were measured in mock and HDV-infected HepaRG^NTCP^ cells. A representative experiment (left panel, mean +/− SEM) and relative OCR (right panel, mean +/− SD, normalized to basal OCR in mock cells, *n* = 5) are shown. **C** OCR was measured in mock and HDV-infected HepaRG^NTCP^ cells treated or not with 1 μM rux for the last 4 days of infection, normalized to basal OCR in mock cells, *n* = 2. Statistical analysis: for **A**, (**B**, right panel) and **C** data are shown as mean +/− SD. For (B, left panel) data are shown as mean +/−SEM. For (A) Mann-Whitney or t-test were used. For (B, right panel and C) one-way ANOVA with Dunn’s multiple comparison test was used. ****p < 0.0001; ***p < 0.001; **p < 0.01; *p < 0.05.

To evaluate the activity of the TCA cycle in more detail, uniformly ^13^C-labelled glucose or glutamine was added to HepaRG^NTCP^ cultures and isotopes were traced (Fig. 4A). Upon HDV infection the fractions of ^13^C carbon for several metabolites such as malate, citrate, succinate and αketoglutarate (αKG), derived from glucose and glutamine tracers were generally lower, confirming reduced activity of the TCA cycle (av. by 25%) (Fig. 4A). In line with the reduced isotope tracing data, the total amount of NADH, a product of the TCA cycle, was reduced in HDV-infected cultures (Fig. S2A). Consistent with reduced availability of NADH and succinate, which are key substrates for oxidative phosphorylation, HDV reduced basal oxygen consumption rates (OCR) ~ by 30% (Fig. 4B), and almost completely eliminated spare respiratory capacity (SRC) (Fig. 4B), while the extracellular acidification rate, a measure of glycolytic activity, was not altered (Fig. S2B). The effects of HDV on respiration were at least partially reversed in the presence of rux (Fig. 4C**)**. Furthermore, recombinant IFN-β also reduced SRC in a similar fashion **(**Fig. S2C**)** suggesting that the effects of HDV on TCA cycle activity are in great part IFN mediated. Staining with mitochondria-specific dyes did not reveal significant differences in mass and structure or the membrane potential of mitochondria (Fig. S2D,E). The total expression levels of the ETC complexes were not altered either (Fig. S2F), suggesting that reduced availability of TCA cycle-derived substrates for oxidative phosphorylation results in decreased basal respiration and SRC.

Pathways contributing most to TCA cycle activity are glycolysis, FAO, and glutaminolysis. Levels of AMPK phosphorylation, one of the key regulators of FAO, remained unaffected (Fig. S2G). Glutaminolysis seemed also unlikely to be involved as isotope tracing using ^13^C-labelled glutamine did not reveal reduction of its activity (data not shown). While PDH activity was not altered by HDV/IFN (Fig. S1B), PC expression was downregulated (Fig. S1C). Thus, changes to pyruvate import cannot be excluded.

In summary, these results show that HDV-induced interferon signalling may lead to metabolic stress related to inhibition of TCA cycle activity that is accompanied by reduced basal respiration and abolished ability of cells to increase respiration.

### Metabolic stress response factors ATF4 and mTORC1 are activated downstream of IFN-β production

**Fig. 5:**
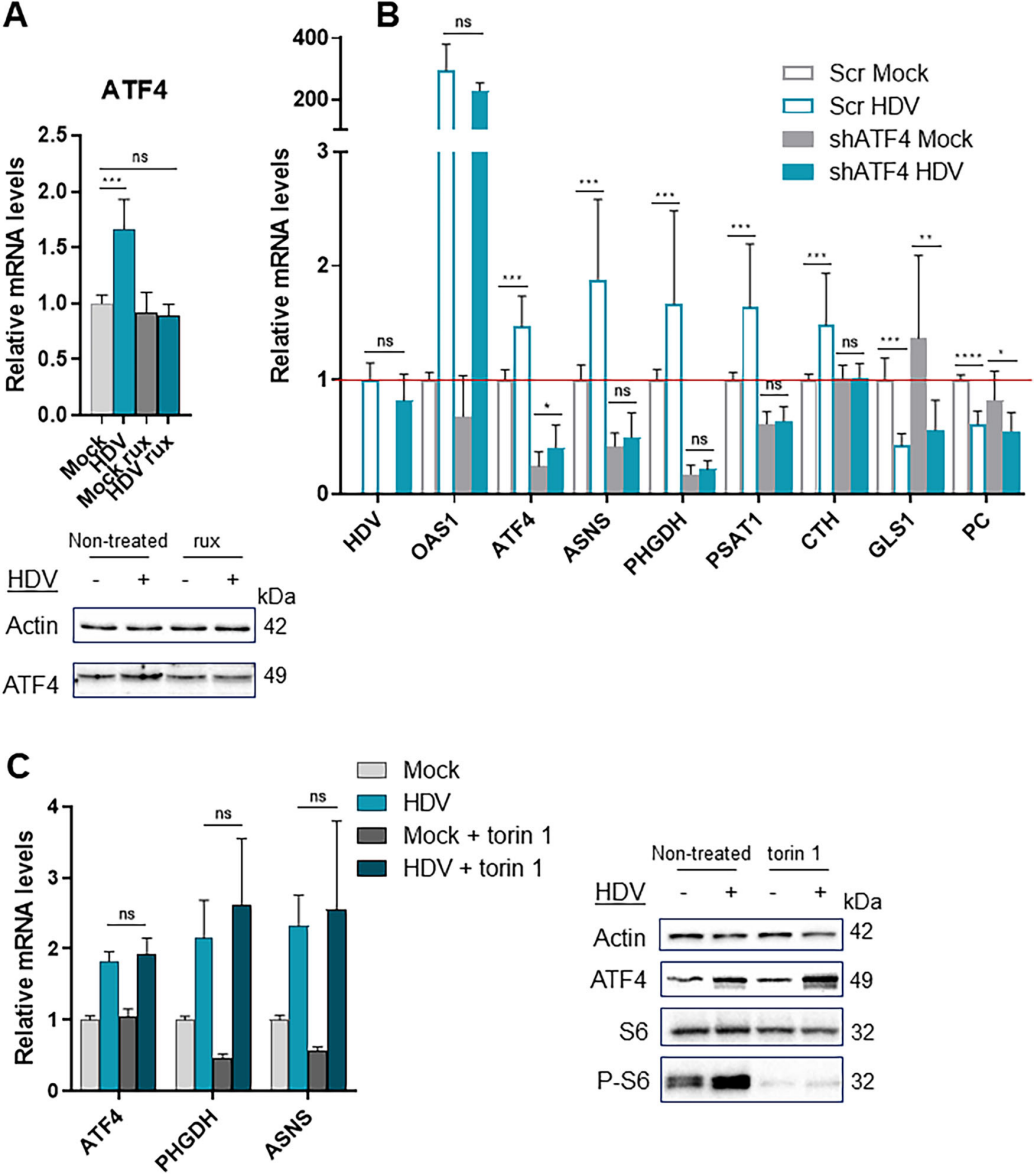
HDV-mediated IFN-β production independently activates metabolic factors ATF4 and mTORC1. **A** Relative mRNA (top panel) and protein (bottom panel) levels in HepaRG^NTCP^ cells treated or not with 1 μM rux for the last 4 days of infection. Normalized to non-treated mock cells, *n* = 3. The Actin panel of the immunoblot is also used in Fig. S2E. **B** Relative mRNA levels in shScrambled (Scr) and shATF4 HepaRG^NTCP^ cells infected or not with HDV. Normalized to scrambled mock cells, *n* = 5. **C** Relative mRNA (left panel) and protein (right panel) levels in HepaRG^NTCP^ cells treated or not with 200 nM of torin 1 for the last 24 h of infection. Normalized to non-treated mock cells, *n* = 3. The non-treated panel of the immunoblot is also used in Fig. S1C and Fig. 2A. Statistical analysis: data are shown as mean +/− SD. For **A**–**C** Mann-Whitney or t-test or one-way ANOVA or one-way ANOVA on ranks with Tukey multiple comparison test were used. ****p < 0.0001; ***p < 0.001; **p < 0.01; *p < 0.05.

Amongst the IFN-target genes that were identified, many are well-known targets of the ATF4 transcription factor as for example those related to serine, asparagine and GSH metabolism [19]. ATF4 is a master regulator of the integrated stress response (ISR) and is implicated in life-death decisions during many types of stress. Indeed, ATF4 mRNA and protein levels were increased in HDV-infected cultures in an IFN-dependent manner **(**Fig. 5A**)**. To investigate a potential role of ATF4 in the metabolic changes induced by HDV/IFN-β, ATF4 expression was silenced using shRNAs **(**Figs. 5B, S3A**)**. ATF4 knock-down did not affect HDV replication or expression of IFN-dependent OAS1 **(**Fig. 5B**)**. However, upregulation of PHGDH, ASNS and CTH was confirmed to be ATF4-dependent, while reduction of GLS1 and PC mRNA levels seemed to involve another mechanism. The best-known mechanism of ATF4 induction is phosphorylation-driven inactivation of eukaryotic translation initiation factor 2α (eIF2α), leading to the initiation of ATF4 translation **(**Fig. S3B**)**. eIF2α mediates stress responses in the settings of unfolded protein accumulation, dsRNA detection, dysregulation of heavy metal metabolism, and amino acid starvation. The type of stress defines which kinase phosphorylates eIF2α. However, neither inhibitors of PKR (C16), PERK (GSK2606414), eIF2α (ISRIB), nor the addition of essential and non-essential amino acids to block GCN2 changed the induction of ATF4 targets PHGDH and ASNS in HDV-infected cultures **(**Fig. S3C, D**)**. Another known ATF4 regulator is the mTORC1 pathway. This pathway was found activated in HDV-infected cells (S6 phosphorylation, Fig. 5C), however, mTORC1 inhibitor torin 1 did not abolish ATF4 induction, excluding this pathway as a regulator of ATF4 **(**Fig. 5C**)**.

In summary, HDV-associated metabolic changes occurring downstream of IFN-β production led to metabolic stress and activation of pro-survival metabolic factors ATF4 and mTORC1. ATF4 activation is mTORC1-independent, and another known ATF4 regulator, eIF2α is also not involved in the IFN-β/ATF4 axis, suggesting induction of ATF4 in this setting by a yet unknown mechanism.

### HDV-induced IFN-β signalling increases the resistance to oxidant-induced cell death and reduces functions specific for differentiated hepatocytes

**Fig. 6:**
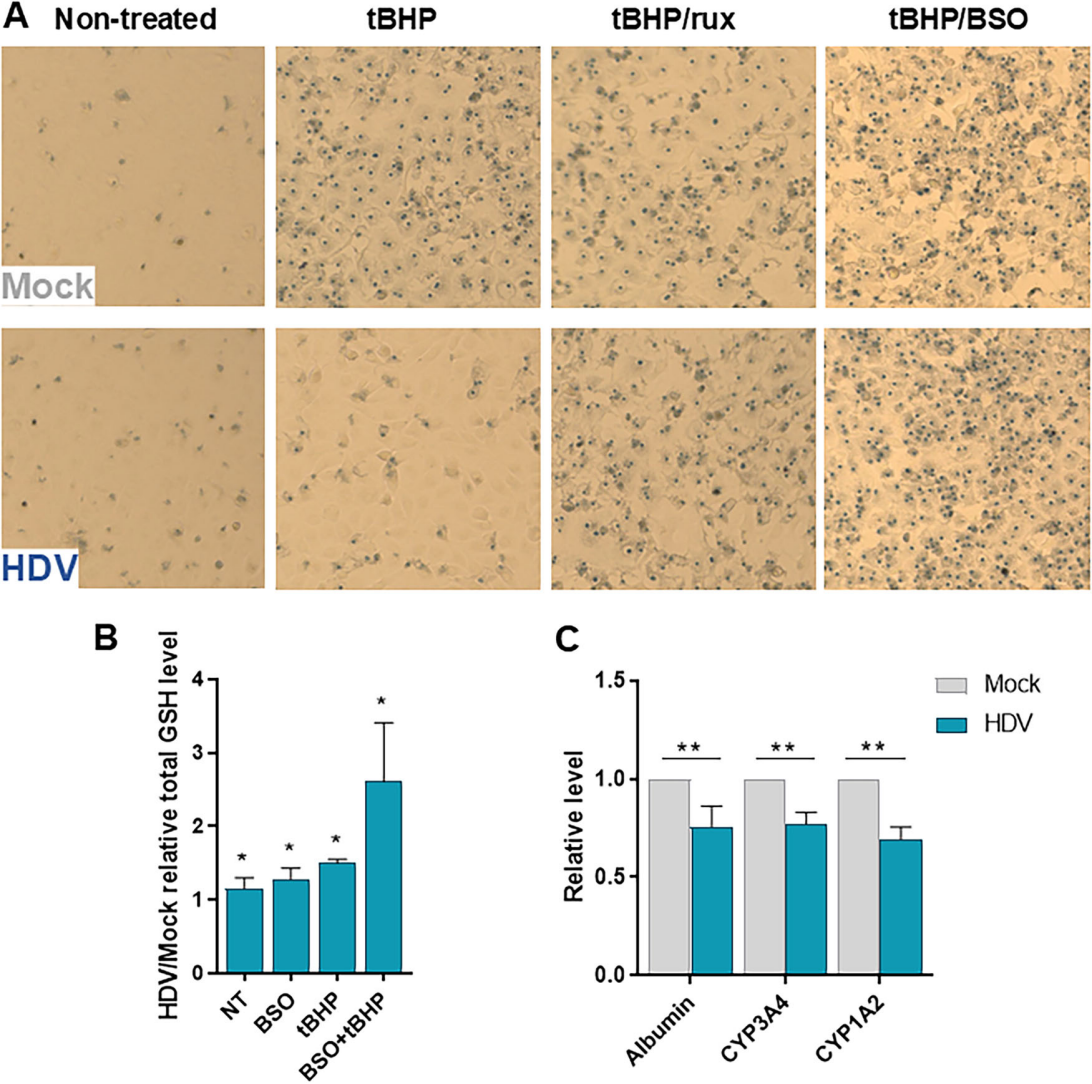
HDV infection renders cells resistant to oxidants and reduces functionality. **A** Trypan blue staining of mock and HDV-infected HepaRG^NTCP^ cells treated or not with 200 μM tBHP for 24 hours; 1 μM rux for 4 days; 10 μM BSO for 29 hours. **B** HDV/mock ratio of total glutathione (GSH) levels. HDV-infected HepaRG^NTCP^ cells pre-treated or not with 10 μM BSO for 5 hours, and then treated or not with 200 μM tBHP for 2 hours. Normalized to non-treated mock cells, *n* = 4. **C** Relative albumin secretion (*n* = 6); CYP3A4 activity (*n* = 3); CYP1A2 activity (*n* = 3) in HepaRG^NTCP^ cells infected with HDV. Normalized to mock cells. Statistical analysis: for **B** and **C** data are shown as mean +/− SD. For **B** and Albumin Mann-Whitney test was used. For CYP3A4 and CYP1A2 t-test was used. ***p < 0.001; **p < 0.01; *p < 0.05.

Based on the finding that GSH levels were induced by HDV/IFN, and ATF4 being a stress response factor, we explored whether ATF4 induction had phenotypic effects, such as protection from stress stimuli and in particular, oxidative stress. HDV significantly increased the resistance to toxic effects of oxidants such as hydrogen peroxide (H_2_O_2_), tert-butyl hydroperoxide (tBHP) and cumene hydroperoxide (data not shown; Fig. 6A, tBHP data). Trypan blue staining was used as a readout for cell viability in order to avoid approaches that may be impacted by the HDV/IFN-induced metabolic changes, such as neutral red uptake or LDH release. The resistance of HDV-infected cells to oxidants was found to be IFN/ATF4-mediated, as the protective effects were reversed by rux treatment (Fig. 6A). Compounds inducing redox imbalance, such as ETC inhibitors rotenone, sodium azide and oligomycin A, or drugs triggering genotoxic and oxidative stress, such as doxorubicin, were equally toxic for mock and HDV-infected cells (Fig. S4A).

To reveal the mechanism underlying the resistance to oxidative stress the levels of key antioxidant molecules GSH and GSSG were measured (Figs. 6B, S4B). Both, GSH and GSSG levels were higher in HDV-infected cells pointing to increased synthesis and oxidation. Pre-treatment with the inhibitor of GSH synthesis BSO restored the sensitivity of HDV-infected cells to tBHP (Fig. 6A). Importantly, treatment with BSO or tBHP slightly increased the GSH ratio between HDV/mock, while the combined treatment BSO + tBHP further aggravated the effect (Fig. 6B). Thus, the IFN response rendered HDV-infected cells resistant to oxidative stress. To check if this was a response to a shifted redox balance, intracellular reactive oxygen species were measured. A small decrease in DCFH2DA fluorescence in HDV-infected cells was detected using flow cytometry (Fig. S4C). However, based on the transcriptome analysis, levels of ROS scavenging enzymes (peroxiredoxins, glutathione peroxidases, catalase) were also slightly reduced. As the redox balance is of particular importance in mitochondria, superoxide anion production was measured in this organelle using MitoSOX dye (Fig. S4C). A slight, but reproducible reduction in superoxide production in mitochondria of HDV-infected cells was detected. Since addition of BSO did not reverse the effect (Fig. S4C), this observation may reflect reduced respiration activity and not increased GSH synthesis. Levels of NADP^+^/NADPH, crucial for mitochondrial antioxidant enzyme recycling, were not altered (Fig. S4D). The Nrf2 pathway did not seem to be involved either, as the Nrf2-transcriptional targets *Nqo1* and *HO1* were not induced (Fig. S4E). Therefore, the resistance of HDV-infected hepatocytes to pro-oxidants likely results from increased GSH biosynthesis.

As an increase of the GSH pool is known to accompany hepatocyte proliferation [20], the differentiation status of HDV-infected cultures was assessed. Infected hepatocyte cultures secreted approximately 25% less albumin into the supernatant (Fig. 6C) and activities of cytochromes CYP3A4 and CYP1A2 were reduced (Fig. 6C). De-differentiation was confirmed in HDV-infected PHH by measuring CYP2C9 activity and mRNA levels of several hepatocyte markers (Fig. S4F, G).

Taken together, IFN-β production upon HDV infection increased GSH synthesis and led to a significant resistance to oxidant-induced toxicity. Moreover, the ability of cultures to maintain key physiological functions – such as albumin secretion and cytochrome activity – was reduced.

## Discussion

Most studies on HDV host cell interactions so far have used transformed hepatoma HepG2^NTCP^ cells. Here, we used non-transformed, differentiated HepaRG^NTCP^ cells, with a metabolic phenotype close to that of PHH [21]. Furthermore, PHH were used to validate effects on hepatocyte-specific differentiation markers, but not for metabolic read outs due to high batch variability, that may either be due to pre-existing liver diseases of donor patients before resection, or - in case of patients with colon cancer metastases in the liver - due to chemotherapy that was applied prior to liver resection. Furthermore, HDV was introduced by its normal infection route instead of the transfection of HDV encoding plasmids. Considering the scarce information available on HDV-host cell interactions, screens were set up to look for changes induced by HDV mono-infection to hepatic metabolism and functions. As previously reported [22], the percentage of HepaRG^NTCP^ cells susceptible to HDV was low, with ca 5%, while the number of HDV-infected hepatocytes in infected patients remains unknown. Consistent with the low infection rate, basically all changes observed in HDV cultures were found to be mediated by IFN-β.

There is surprisingly little information available on the effect of type I interferons on the metabolism of hepatocytes. In primary mouse hepatocytes treated with IFN-β for 24 h, a reduction of mRNA expression of genes involved in GSH, amino acid, drug, fatty acid and cofactor metabolism has been reported [1]. In our hands, HDV-infected HepaRG^NTCP^ cultures showed a decreased expression of fatty acid metabolism-related genes, but synthesis of asparagine, serine and GSH was activated at the transcript, protein and metabolite levels. This contradiction with the literature on IFN-β mediated metabolic effects may be explained by time- and model-dependent effects. To reveal the mechanism of IFN-β-induced amino acid synthesis, regulation by the mTOR pathway was analysed, as it has been reported to be activated by type I interferons in osteosarcoma and myeloma cell lines [23]. It was indeed activated, but its inhibition did not reverse the observed metabolic events. Furthermore, a panel of upregulated genes (ASNS, PHGDH, PSAT1, CTH) is known to be ATF4 targets [19]. The ATF4 pathway was activated in HDV-infected cultures. ATF4 knockdown confirmed that amino acid metabolism activation is ATF4-dependent downstream of IFN-β signalling. ATF4 is a stress-response pathway able to induce pro- and anti-survival programmes [24]. ATF4 regulation by eIF2α in the context of unfolded protein response (UPR) is well described [25]. However, little is known about ATF4 activation by type I IFNs. A study in beta cells showed induction of the UPR by IFN-α [26]. While the ATF4 pathway is not discussed in this report, it is frequently activated downstream of UPR. Another report described mutual regulation between IFN and ATF4 involving IRF7 [27]. However, eIF2α was not responsible for ATF4 activation in HDV-infected cultures. It was also reported that mitochondrial dysfunction can trigger ATF4, and thus, in our case, its activation could be secondary to IFN-β-mediated disturbance of the TCA cycle and respiratory activity [28]. Taken together, HDV-mediated IFN-β production and signalling orchestrate a rewiring of the hepatocyte metabolic phenotype. ATF4 is activated downstream of IFN-β by yet unknown eIF2α- and mTOR-independent mechanisms.

The next step was to try to understand the consequences of the observed IFN-β-mediated changes. An increased activity of amino acid and GSH synthesis, accompanied by a decrease of TCA cycle activity and respiration in HDV-infected cultures, did not initially paint a clear picture about whether these changes were pro- or anti-viral. Nutrient limitation or pharmacological inhibition of core metabolic pathways (glycolysis, glutaminolysis, purine synthesis) did not reveal any impact on HDV replication. Neither were HDV-infected cultures vulnerable to glucose and/or glutamine and/or serum removal (data not shown). Moreover, a significant IFN-β-mediated resistance of HDV-infected cultures to oxidants was found and is likely due to increased GSH synthesis, as the protection was lost in the presence of the GSH synthesis inhibitor BSO. The BSO target enzyme, glutamate-cysteine ligase (GCL), protects the cell from ferroptosis induced by glutamate accumulation during cystine starvation [29]. However, it is more likely that cytotoxicity is a direct effect of tBHP rather than it being secondary to tBHP-mediated cystine depletion. In the liver, ROS production takes place during drug and toxin catabolism [30]. It also accompanies inflammatory processes [31], which are common in HDV-infected liver tissue. The increased GSH synthesis may alleviate tissue damage in an oxidative environment, but in the long term may favour survival of infected cells, and consequently cancer initiation and progression [32]. Furthermore, differentiated hepatocytes ensure highly specialized secretory, metabolic and detoxification processes. Type I IFNs have previously been reported to reduce cytochrome activity [33]. Consistently, we observed reduced albumin secretion and CYP3A4 and CYP1A2 activities in HDV-infected HepaRG^NTCP^ cultures and confirmed reduced CYP2C9 activity and reduced transcript levels of differentiation markers and genes involved in FAO in HDV-infected PHH, which is in line with published RNAseq data [34]. However, changes to albumin secretion did not reach statistical significance in PHH. The fact that the PHH used here were derived from donor patients with liver metastases due to colon cancer, who underwent chemotherapy and 4 weeks later liver resection, may explain these discrepancies. Whether the observed loss of differentiation factors is associated with liver function loss and increased proliferation and whether increased proliferation may impact HDV persistence or spread, as previously reported [22], warrants further investigation. Indeed, both HDV-induced and exogenous IFN have been shown to inhibit HDV in dividing cells [22]. However, in our differentiated cultures, neither endogenous nor exogenous IFN impacted HDV replication (Figs. 1A, 2A–C).

Besides affecting cellular differentiation, the IFN-mediated increase in GSH levels may preserve a cysteine residue in L-HDAg, known to be crucial for viral spread, from oxidation, and to favour cell survival. While the presented dataset needs to be validated in patient tissues, increased cell survival in the context of the inflammatory, oxidative and fibrotic microenvironment observed in many CHD patients may favour and drive the associated liver pathology. In conclusion, a more detailed understanding of how IFN-induced metabolic changes impact viral infections and contribute to their pathological sequelae is needed in order to optimize therapeutic strategies.

## Materials and Methods

### For details of reagents used, please see supplementary materials

#### Cell culture

Cells were grown in a 5% CO_2_ humidified atmosphere at 37 °C. HepaRG, HepaRG^NTCP^ and PHH cells were cultured in William’s E medium containing GlutaMAX, 5 µg/mL human insulin, 50 µM hydrocortisone hemisuccinate, 100 IU/mL penicillin, 100 µg/mL streptomycin, and 10% fetal bovine serum for HepaRG cells and 5% for PHH (Fetal clone II). HepaRG^NTCP^ cell culture medium was supplemented with 5 µg/mL blasticidin and 100 µM zeocin. Medium was renewed twice a week. Cells were split once a week till passage 16-18. PHH were seeded on plates covered with collagen type I and used within 2 weeks after seeding.

### HDV infection of HepaRG and PHH cells

HepaRG cells were seeded at a sub-confluent density. After two weeks of culture, the medium was supplemented with 1.8% DMSO, and the cells were left for two more weeks with medium changes twice a week. 4 weeks post-seeding, cells were infected with HDV at an MOI 10 in the presence of DMSO and 4% PEG 8000 for 24 h. Mock cells were treated only with PEG and DMSO. Then cells were washed with PBS and incubated in the medium with DMSO. HepaRG^NTCP^ cells were seeded at a sub-confluent density. After 4–5 days of culture, the medium was supplemented with 1.8% DMSO and 1 µg/mL tetracycline. HDV infection was done on day 7-8 post-seeding, equally to the HepaRG cell line. All infections were performed in duplicates or triplicates. PHH cells were maintained in the medium supplemented with 2% DMSO and infected with HDV at an MOI 30 as described above for HepaRG cells.

### Mito stress and glycolysis stress tests

HepaRG^NTCP^ cells were seeded in 24-well SeaHorse plates and infected with HDV for 7 days as described above (5 replicates). 24 hours prior to analysis, the cartridge was soaked in Seahorse XF Calibrant Solution. Thirty minutes before the analysis, the medium was replaced with Seahorse XF DMEM supplemented with 2 mM glutamine and 11 mM glucose (glucose was added only for the mito stress test). For the Mitostress test, oxygen consumption rate was measured first without any supplementation (basal respiration), then cells were treated subsequently with 1 μM oligomycin A (ATP-dependent respiration), 0.75 μM FCCP (spare respiratory capacity), and a mixture of 1 μM rotenone and 1 μM antimycin A (non-mitochondrial oxygen consumption). For the glycolysis stress test, extracellular acidification rate was measured first without any supplementation (basal glycolytic activity), then cells were treated subsequently with 11 and 30 mM glucose, 1 μM oligomycin A, 50 mM 2-deoxyglucose. Data acquisition was performed with the Seahorse XFe24 Extracellular Flux Analyzer (Agilent, CA, USA).

### qPCR

Quantitative PCR was performed on QuantStudio 7 Flex (Applied Biosystems, MS, USA) using 2 µL of cDNA per reaction, and reactions were performed in duplicates. The programme included the following steps: pre-incubation step of 95 °C for 5 min, then 40 cycles of 95 °C/10 sec; 55 °C/20 sec; 72 °C/20 sec, then melting step 95 °C/5 sec; 65°C/1 sec; 97 °C/1 sec. GUS (β-glucuronidase) was used as a housekeeping gene.

### Cytotoxicity assay (trypan blue)

#### Trypan blue

HepaRG^NTCP^ cells were seeded in 24-wells. Cell medium was removed, cells were washed once with PBS and incubated with 0.2% trypan blue solution in PBS for 5 min. Then staining solution was replaced by PBS. Cells were visualized using the EVOS FL Auto Imaging System (Thermo Scientific, MS, USA).

### NAD^+^/NADH, NADP^+^/NADPH and GSH/GSSG measurement

HepaRG^NTCP^ cells were seeded in 48-well plates and infected with HDV for 7 days as described above. Metabolite measurements were performed according to the manufacturer’s protocols. Signals were detected using a Luminoskan (Thermo Scientific, MS, USA).

### Cytochromes CYP3A4, CYP1A2 and CYP2C9 activity

HepaRG^NTCP^ or PHH cells were seeded in 48-well plates and infected with HDV for 7 days as described above. CYP3A4, CYP1A2 and CYP2C9 activities were measured according to the manufacturer’s protocol. Cells were incubated with CYP3A4 or CYP2C9 substrate overnight to increase sensitivity. Signals were detected using a Luminoskan.

### Conditioned medium dialysis

HepaRG^NTCP^ cells were infected with HDV for 7 days as described above. The conditioned medium was collected, filtered through a 0.2 µm filter (PES membrane) and loaded into the dialysis cassette (Slide-A-Lyzer 2 K MWCO, ThermoFisher, MS, USA). The dialysis was performed at +4 °C for 12 hours against 20x volume of complete cell culture medium without DMSO with a single medium change after 6 hours. The resulting dialyzed conditioned medium was supplemented with 1.8% DMSO and incubated with HepaRG^NTCP^ cells for 3 days.

### IFN-β neutralization with antibodies

HepaRG^NTCP^ cells were infected with HDV as described above. The next day after infection, anti-IFN-β neutralizing antibody was added at a concentration 1 µg/mL. Every second day, a fresh aliquot of the antibody was added without medium change. Anti-Hepatitis B virus core antigen antibodies were used as an isotypic control.

### Quantification and statistical analysis

Data are represented as means ± SD or SEM. Graphical illustrations and significance were obtained with GraphPad Prism 7 (Graph-Pad) and SigmaPlot 12.5, respectively. The statistical test used in each quantification is reported in the Figure legend. The levels of significance were set as *p < 0.05; **p < 0.01; ***p < 0.001; ****p < 0.0001.

## Supplementary information

Supplementary Figures and Materials and Methods

Original immunoblots

## Data Availability

Further information and requests for resources and reagents should be directed to and will be fulfilled by the Lead Contact, Birke Bartosch (birke.bartosch@inserm.fr).
