# Supplementary material for: IFN-β production promotes metabolic rewiring and protection against oxidative stress in hepatitis delta virus-infected hepatocyte cultures
Source: Cell Death Dis. 2025 Jul 18;16(1):534. doi: 10.1038/s41419-025-07838-z (PMC12274286; doi:10.1038/s41419-025-07838-z)
Supplement: Supplementary file 1 — Supplementary Figures and Materials and Methods [file 41419_2025_7838_MOESM1_ESM.pdf]

Supplementary Figure 1

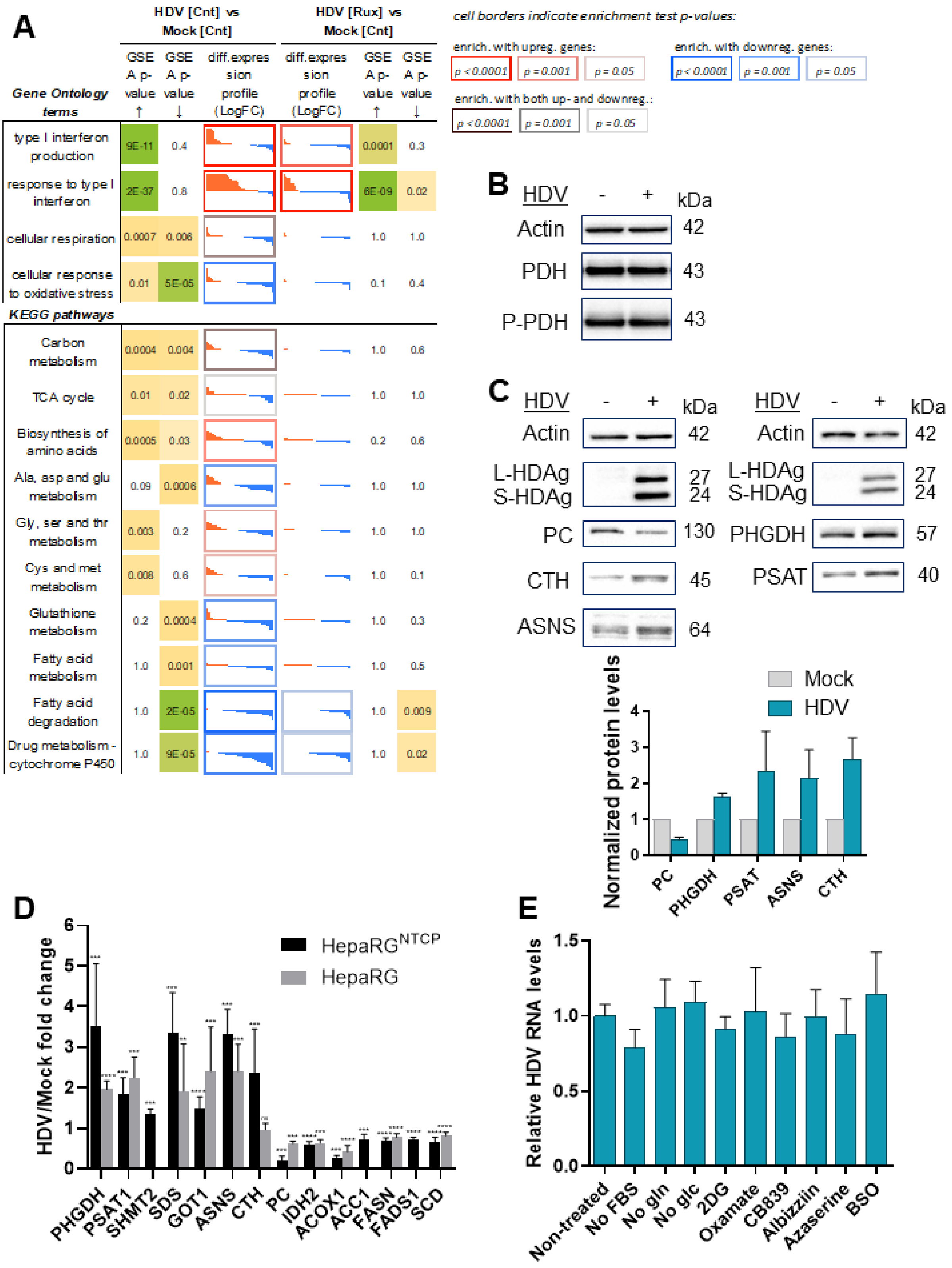

Fig. S1. Impact of HDV on expression profiles of metabolic genes and HDV sensitivity to metabolic inhibitors.

(A) Differential expression profiles of either upregulated (red) or downregulated (blue) GO or KEGG pathways in HDV-infected cells (treated or not with rux) compared to untreated and non-infected cells. Relative expression values are log2-transformed. Genes are sorted by fold change in expression level in a decreasing order. Vertical axis range in each cell is from -2 (i.e. 4-fold expression increase) to +2 (i.e. 4-fold expression decrease). The colour code of the cell borders indicates the level of significance of enrichment.
(B) Representative immunoblot analysis of mock and HDV-infected HepaRGNTCP cells. n = 3
(C) Immunoblot analysis (upper panel) of mock and HDV-infected HepaRGNTCP cells and the corresponding relative quantification (lower panel, n = 3). The right panel of the immunoblot is also used in Fig. 2A and Fig. 4D as non-treated controls.
(D) HDV/mock ratio of mRNA levels in HepaRGNTCP and HepaRG cells infected with HDV for 7 days. n = 3-5.
(E) HDV RNA levels in HDV-infected HepaRGNTCP cells treated as indicated for the last 4 days of infection (drug concentrations are detailed in the Materials and methods section). Normalized to non-treated cells, n = 2.
Statistical analysis: for (C-E) data are shown as mean +/- SD. For (D) Mann-Whitney or t-test were used. For (E) one-way ANOVA was used, all treated vs non-treated differences are non-significant. ****: $p < 0.0001$; ***: $p < 0.001$.

Supplementary Figure 2

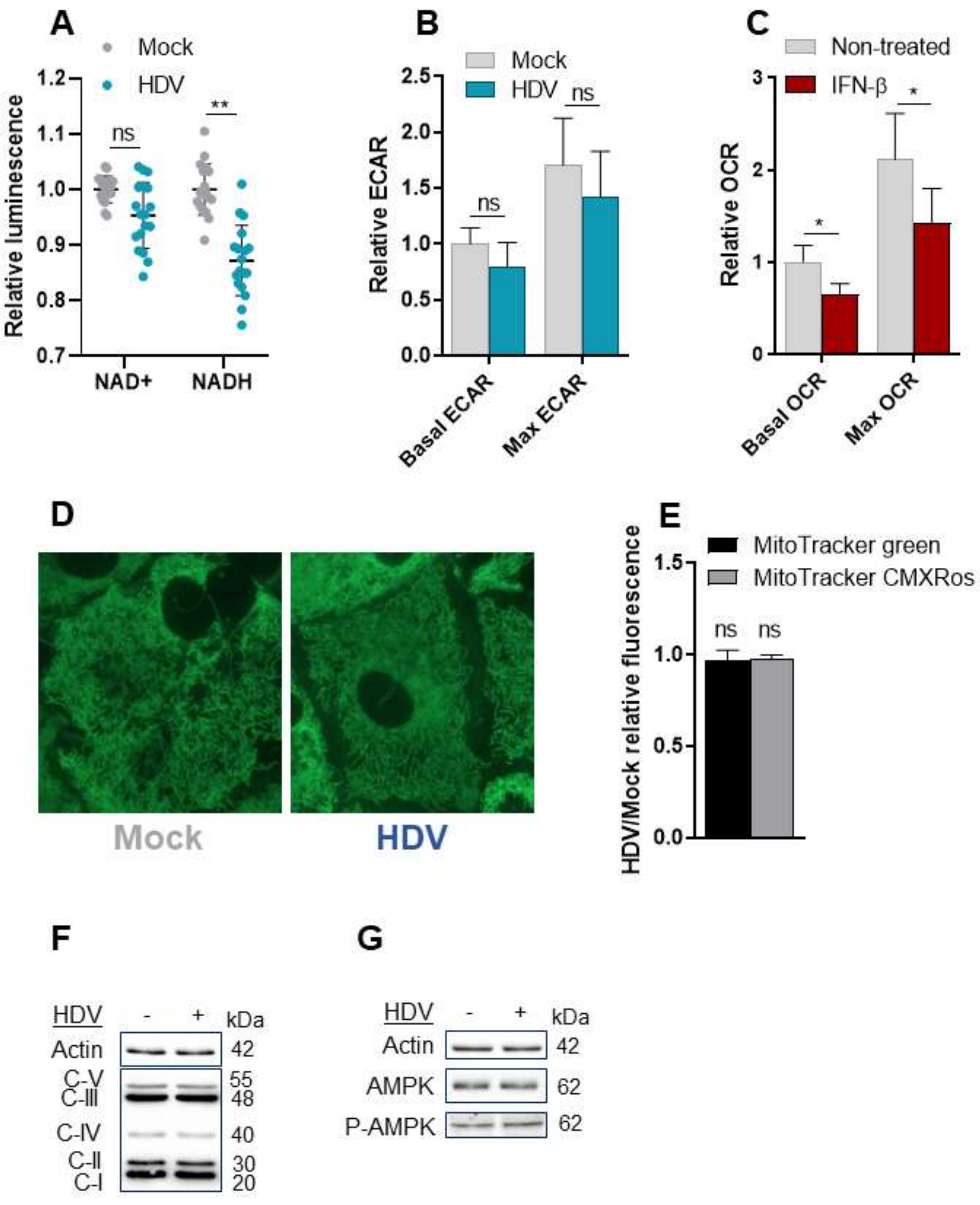

Fig. S2. HDV's impact on mitochondrial functions.

(A) Relative NAD^+ and NADH levels in $HepaRG^{NTCP}$ cells infected with HDV, n = 6.

(B) Extracellular acidification rates (ECAR) were measured in mock and HDV-infected $HepaRG^{NTCP}$ cells. Normalized to basal ECAR in mock cells, n = 2.

(C) OCR was measured in naïve HepaRGNTCP cells treated or not with 1 ng/mL of IFN-β for 3 days, normalized to basal OCR in non-treated cells, n = 2.

(D) Live MitoTracker green FM staining of $HepaRG^{NTCP}$ cells infected or not with HDV.

(E) Relative MitoTracker green FM and MitoTracker CMXRos fluorescence measured by flow cytometry in HepaRG^{NTCP} cells infected with HDV, n = 3.
(F) Representative immunoblot analysis of respiratory complexes I–V expression in mock and HDV-infected HepaRG^{NTCP} cells. The Actin panel of the immunoblot is also used in Fig. 4A as the non-treated control. n = 3
(G) Representative immunoblot analysis of AMPK and P-AMPK expression in mock and HDV-infected HepaRG^{NTCP} cells, n = 2
Statistical analysis: for (A), (B), (C) and (D), data are shown as mean +/- SD. For (A) Mann-Whitney test was used. For (B) and (C) one-way ANOVA was used, all mock vs HDV differences are non-significant (ns). For (D) t-test was used. **: $p < 0.01$.

Supplementary Figure 3

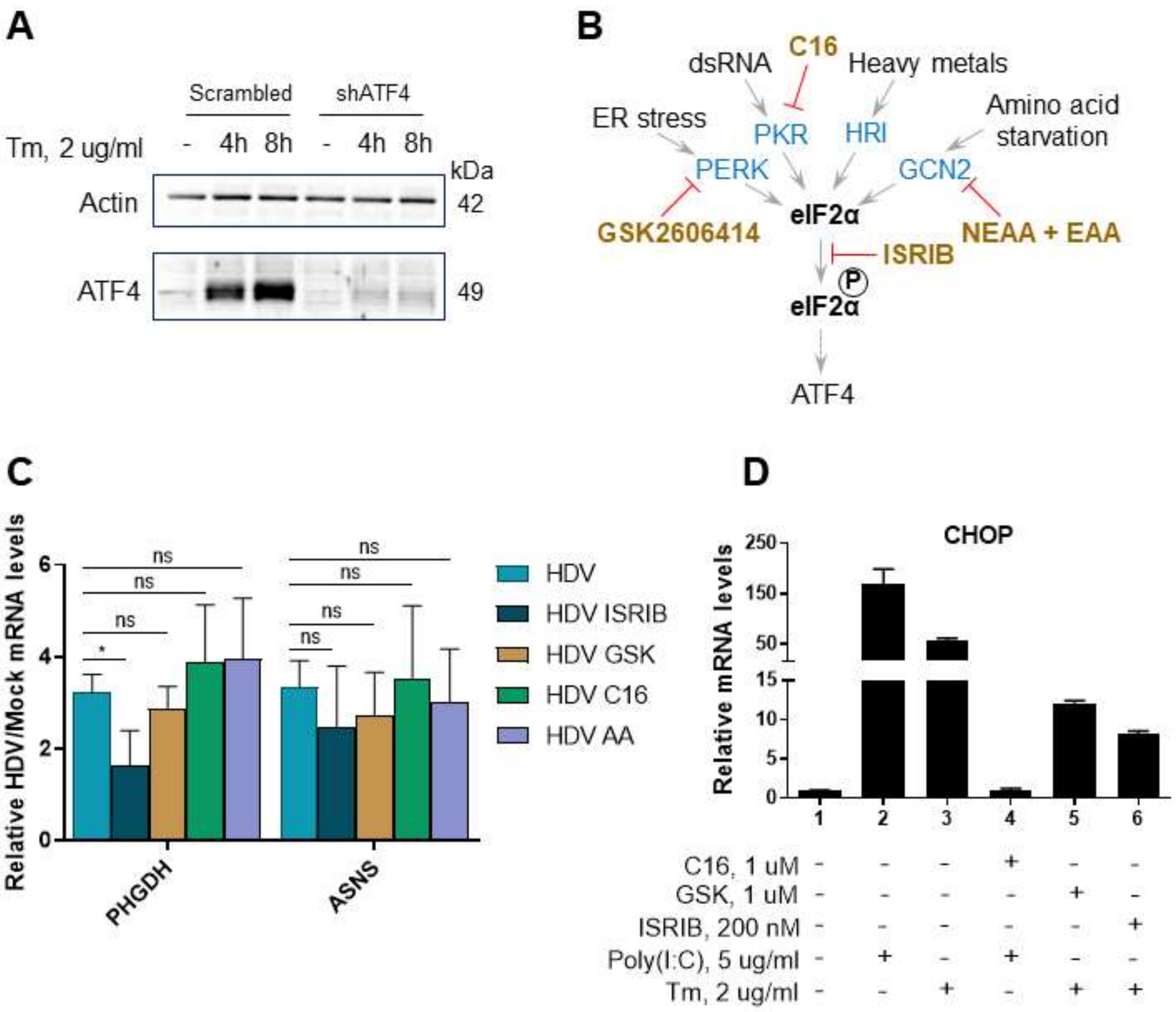

Fig. S3. ATF4 activates serine and asparagine synthesis downstream of IFN-β signalling

(A) Immunoblot analysis of shScrambled and shATF4 HepaRGNTCP cells treated or not with tunicamycin (Tm) for the indicated times.

(B) eIF2α-mediated ATF4 regulation

(C) HDV/mock ratio of mRNA levels in HepaRGNTCP cells treated with ISRIB (200 nM), GSK2606414 (1 µM), C16 (1 µM), or NEAA + EAA for the last 4 days of HDV infection. Normalized to non-treated mock cells, n = 3.

(D) Relative *CHOP* mRNA level in HepaRG^{NTCP} cells treated with ISRIB (200 nM), GSK2606414 (1 µM), C16 (1 µM) in the presence or absence of tunicamycin (Tm, 2 µg/mL) or transfected poly(I:C) (5 µg/mL). Normalized to non-treated cells, n = 2.

Statistical analysis: data are shown as mean +/- SD. For (B) one-way ANOVA with Dunnett multiple comparison test was used. *: $p < 0.05$.

Supplementary Figure 4

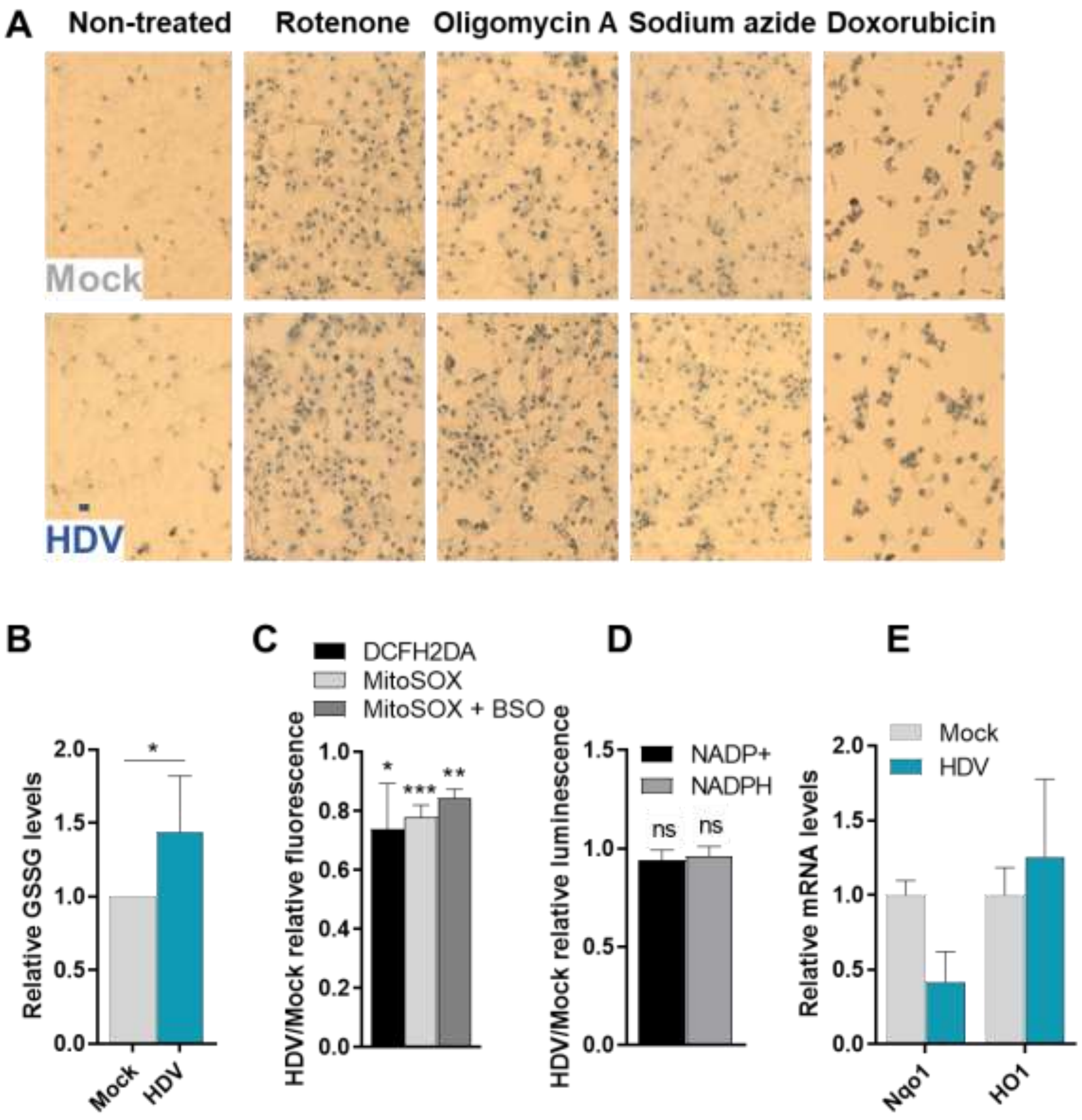

Fig. S4. HDV infection increased both GSSG and GSH synthesis, but did not alter $NADP^+$ and NADPH production.

(A) Trypan blue staining of mock and HDV-infected HepaRG^{NTCP} cells treated or not with 0.5 µM rotenone for 2 days; 0.25 µM oligomycin A for 2 days; 0.25 mM sodium azide for 2 days; 3 µg/mL doxorubicin for 3 days.

(B) HDV/mock ratio of GSSG levels in HepaRG^{NTCP} cells infected with HDV. Normalized to mock cells, n = 2.

(C) HDV/mock ratio of DCFH2DA and MitoSOX fluorescence in HepaRG^{NTCP} cells infected with HDV. Normalized to mock cells, n = 3 (DCFH2DA, MitoSOX), n = 2 (MitoSOX + BSO).

(D) Relative $NADP^+$ and NADPH levels in HepaRG^{NTCP} cells infected with HDV. Normalized to mock cells, n = 3.

(E) Relative mRNA levels in HepaRG^{NTCP} cells. Normalized to mock cells, n = 4.

Statistical analysis: for (B-F) data are shown as mean +/- SD. For (B) Mann-Whitney test was used. For (C), (E) and (F) t-test was used. ***: $p < 0.001$; **: $p < 0.01$; *: $p < 0.05$.

SUPPLEMENTARY MATERIALS AND METHODS

REAGENT	SOURCE	IDENTIFIER
**Antibodies**		
Anti-PHGDH	Merck	Cat.# HPA021241-100UL
Anti-ATF4	Cell Signaling	Cat.# 11815S
Anti-CTH	Merck	Cat.# HPA023300-100UL
Anti-PC	Merck	Cat.# HPA058765-100UL
Anti-PSAT	Merck	Cat.# HPA042924-100UL
Anti-ASNS	Merck	Cat.# HPA064737-100UL
Anti-β-Actin	Merck	Cat.# A5316-100UL
Anti-OXPHOS	Abcam	Cat.# ab110411
Anti-HDAg	Alexander Ivanov	Serum 100-3a
Anti-S6	Cell Signaling	Cat.# 2317S
Anti-P-S6	Cell Signaling	Cat.# 2211S
Anti hIFN-β-IgG	Invivogen	Cat.# mabg2-hibnb-3
Anti-HIL-29-IgG	Invivogen	Cat.# mabg-hil29-3
Anti-Hepatitis B Virus Core Antigen	Abcam	Cat.# ab8637
**Cell culture reagents**		
HyClone FetalClone II serum	GE Healthcare	Cat.# SH30066.03
HyClone Fetal bovine serum	Fisher Scientific	Cat.# 12389802
William's E medium	Thermo Fisher Scientific	Cat.# 12551032
DMEM high glucose	Thermo Fisher Scientific	Cat.# 11960044
DMEM no glucose, no glutamine	Thermo Fisher Scientific	Cat.# A1443001
GlutaMAX	Thermo Fisher Scientific	Cat.# 35050061
Penicillin-Streptomycin	Thermo Fisher Scientific	Cat.# 15070063

Trypsin-EDTA (0.05%)	Thermo Fisher Scientific	Cat.# 25300054
DMSO	Merck	Cat.# D2650
Human insulin	Merck	Cat.# I9278
Hydrocortisone hemisuccinate	Merck	Cat.# H2270
Collagen I	Merck	Cat.# CLS354236
**Other reagents**		
Ruxolitinib	Santa Cruz	Cat.# sc-364729
BX795	Santa Cruz	Cat.# sc-281689
IFN beta recombinant	Sino Biological	Cat.# 10704-HNAS
CB839	Calbiochem	Cat.# 5337170001
Protease and phosphatase inhibitors	Thermo Fisher Scientific	Cat.# A32961
PEG 8000	Merck	Cat.# 81268
C16	Merck	Cat.# I9785
ISRIB	Merck	Cat.# SML0843
GSK2606414	Santa Cruz	Cat.# sc-490182
2-deoxyglucose	Santa Cruz	Cat.# sc-202010
Torin 1	Merck	Cat.# 475991
BSO	Merck	Cat.# B2515
Azaserine	Santa Cruz	Cat.# sc-29063
Albizziin	Santa Cruz	Cat.# sc-218627
Triptolide	Santa Cruz	Cat.# sc-200122
Poly (I:C) LMV	Invivogen	Cat.# tlrl-picw
Lipofectamine 2000	Thermo Fisher Scientific	Cat.# 11668030
Sodium oxamate	Merck	Cat.# O2751-5G
Proteinase K	Merck	Cat.# P5568
GlycoBlue	Thermo Fisher Scientific	Cat.# AM9516
Cumene hydroperoxide	Merck	Cat.# 247502-5G

MitoTracker green FM	Thermo Fisher Scientific	Cat.# M7514
MitoTracker Red CMXRos	Thermo Fisher Scientific	Cat.# M7512
MitoSOX	Thermo Fisher Scientific	Cat.# M36008
2′,7′-Dichlorofluorescein diacetate	Santa Cruz	Cat.# sc-209391
Trypan blue solution 0.4%	Merck	Cat.# T8154
Sulforhodamine B (SRB)	Merck	Cat.# 230162
Seahorse XF Cell Mito Stress Test Kit	Agilent	Cat.# 103015-100
Seahorse XF Glycolysis Stress Test Kit	Agilent	Cat.# 103020-100
Seahorse XF DMEM medium, pH 7.4	Agilent	Cat.# 103575-100
Seahorse XFe24 FluxPak	Agilent	Cat.# 102340-100
Seahorse XF Calibrant Solution	Agilent	Cat.# 100840-000
D-Glucose 13C6	EURISO-TOP	Cat.# CLM-1396-1
L-Glutamine 13C5	Cortecnet	Cat.# CC1050P01
Methanol LC-MS grade	Carl Roth	Cat.# AE71.2
Acetonitrile UHPLC-MS grade	Merck	Cat.# 14261-2L
Ammonium carbonate (MS)	Merck	Cat.# 379999-10G
RNAlater solution	Merck	Cat.# R0901-100ml
**Kits**		
Lipofectamine LTX	Thermo Fisher Scientific	Cat.# 15338100
NucleoSpin RNA Virus kit	Macherey-Nagel	Cat.# 740956.50
NAD^+/NADH-Glo assay	Promega	Cat.# G9071
$NADP^+$/NADPH-Glo assay	Promega	Cat.# G9081
GSH/GSSG-Glo assay	Promega	Cat.# V6611
Albumin ELISA	MyBioSource	Cat.# MBS564029-1x96
TRI reagent	MRC	Cat.# TR 118
DNase I	Merck	Cat.# 4716728001
FastStart SYBR Green Mix	Merck	Cat.# 4913914001

M-MLV reverse transcriptase	Invitrogen	Cat.# 28025013
Pierce BCA protein assay kit	Thermo Fisher Scientific	Cat.# 23225
P450-Glo CYP3A4 assay with luciferin-IPA	Promega	Cat.# V9001
P450-Glo CYP1A2 assay	Promega	Cat.# V8421
P450-Glo CYP2C9 assay	Promega	Cat.# V8791
**Plasmids**		
pLP1	Invitrogen	Cat.# K4975-00
pLP2	Invitrogen	Cat.# K4975-00
VSV-G	Invitrogen	Cat.# K4975-00C
LeGO-G/Neo-opt	Lentiviral Gene Ontology Vectors	N/A
pSVLD3	Addgene	Cat.# 29335
pT7HB2.7	Camille Sureau	N/A
pDL444	Addgene	Cat.# 29337
**Experimental models: cell lines**		
HepaRG	(39)	N/A
HepaRG^{NTCP}	Julie Lucifora	N/A
Huh7.5	Charles Rice	N/A
HEK293T	ATCC	CRL-3216
**Oligonucleotides**		
CACATTCTTGGGCTGAAC	Eurogentec	PHGDH mRNA forward
TTATTAGACGGTTATTGCTGTA	Eurogentec	PHGDH mRNA reverse
TGCCGCACTCAGTGTTGTTA	Eurogentec	PSAT1 mRNA forward
GCTAGCAATTCCCGCACAAG	Eurogentec	PSAT1 mRNA reverse
GGGCCCTTGCGGAGTT	Eurogentec	PC mRNA forward
CTGCTGGTTGTTGAGCACATTC	Eurogentec	PC mRNA reverse

CTGCACGCCCTCTATGACA	Eurogentec	ASNS mRNA forward
TAAAAGGCAGCCAATCCTTCT	Eurogentec	ASNS mRNA reverse
AGGTGGTAAAGGGTGGCTCC	Eurogentec	OAS1 mRNA forward
ACAACCAGGTCAGCGTCAGAT	Eurogentec	OAS1 mRNA reverse
GATGTACAGGTTGGCAGATCTCA	Eurogentec	ALDH1L1 mRNA forward
GATGATGATGCCACAAACCCC	Eurogentec	ALDH1L1 mRNA reverse
GCCACGGCTCATCATAGCTG	Eurogentec	SHMT2 mRNA forward
AGCAGGTGTGCTTTGACTTCA	Eurogentec	SHMT2 mRNA reverse
CCAACAACAGCAAGGAGGAT	Eurogentec	ATF4 mRNA forward
GTGTCATCCAACGTGGTCAG	Eurogentec	ATF4 mRNA reverse
TGTCCTCCACTCCAGATCATTTCAA GAGAATGATCTGGAGTGGAGGACT TTTTTC	Eurogentec	ATF4-sh1 forward
TCGAGAAAAAAGTCCTCCACTCCA GATCATTCTCTTGAAATGATCTGGA GTGGAGGACA	Eurogentec	ATF4-sh1 reverse
TGCACTTCAAACCTCATGGGTTCAA GAGACCCATGAGGTTTGAAGTGCT TTTTTC	Eurogentec	ATF4-sh2 forward
TCGAGAAAAAAGCACTTCAAACCT CATGGGTCTCTTGAACCCATGAGG TTTGAAGTGCA	Eurogentec	ATF4-sh2 reverse
TGCTAAGGTTAAGTCGCCCTTTCAA GAGAAGGGCGACTTAACCTTAGCT TTTTTC	Eurogentec	Scrambled forward

TCGAGAAAAAAGCTAAGGTTAAGT CGCCCTTCTCTTGAAAGGGCGACTT AACCTTAGCA	Eurogentec	Scrambled reverse
GACCTCATCAGGTTTGCCCA	Eurogentec	IDH2 mRNA forward
AGTGCTCGTTCAGCTTCACA	Eurogentec	IDH2 mRNA reverse
ATTGATCGGAGGTCTCGGTGT	Eurogentec	IDH3 mRNA forward
CAGGAGGGCTGTGGGATTC	Eurogentec	IDH3 mRNA reverse
AGCTGCTGCAGAGGGTCATGT	Eurogentec	GLS1 KGA mRNA forward
CCACCTGTCCTTGGGGAAAGGGTT	Eurogentec	GLS1 KGA mRNA reverse
CCCTCCTTTCCCTTTTCCGA	Eurogentec	ACOX1 mRNA forward
TCCCAGGCATACCCTCTACA	Eurogentec	ACOX1 mRNA reverse
TACAAGCTGCGTGCCGCTGA	Eurogentec	FASN mRNA forward
ACCCTCGATGACGTGGACGGAT	Eurogentec	FASN mRNA reverse
TTCACTCCACCTTGTCAGCGGA	Eurogentec	ACC1 mRNA forward
GTCAGAGAAGCAGCCCATCACT	Eurogentec	ACC1 mRNA reverse
CATTTTGTGATTGGCCACCT	Eurogentec	FADS1 mRNA forward
GTCTTTGCGGAAGCAGTTG	Eurogentec	FADS1 mRNA reverse
CACTTGGGAGCCCTGTATGG	Eurogentec	SCD mRNA forward
TGAGCTCCTGCTGTTATGCC	Eurogentec	SCD mRNA reverse
GGCTCTACCTGCGTGCTTTA	Eurogentec	CTH mRNA forward
ATTCAAAACCCGAGTGCTGG	Eurogentec	CTH mRNA reverse
ACTGGAGGGGAATCTCCGAA	Eurogentec	SDS mRNA forward
TTTGGCCTCTGCATAGTGGG	Eurogentec	SDS mRNA reverse
CGGGCCGGCTACTCTTCT	Eurogentec	HDAg mRNA forward
AAGGAAGGCCCTCGAGAACA	Eurogentec	HDAg mRNA reverse
CGTGGTTGGAGAGCTCATTTGGAA	Eurogentec	GUS mRNA forward
ATTCCCCAGCACTCTCGTCGGT	Eurogentec	GUS mRNA reverse

AGGCACTGAGCGTATCATGT	Eurogentec	CHOP mRNA forward
TCCTTCTTGAACACTCTCTCCT	Eurogentec	CHOP mRNA reverse
CAGTGCAGGGGAAAGAATAGTAGAC	Eurogentec	pLEGO-seq forward
AAACCTACAGGTGGGGTCTTTC	Eurogentec	pLEGO-seq reverse

HDV infection of HepaRG and PHH cells

For HDV infection HepaRG cells were seeded at the density allowing cells to be sub-confluent the next day. After two weeks of culture, the medium was supplemented with 1.8% DMSO, and the cells were left for two more weeks with the medium change twice a week. 4 weeks post-seeding cells were infected with HDV at MOI 10 in the presence of DMSO and 4% PEG 8000 (stock solution 40% PEG 8000 in PBS, sterile-filtered) for 24h. Mock cells were treated only with PEG and DMSO. Then cells were washed with PBS and incubated in the medium with DMSO. HDV replication reaches the maximum on day 4 and is stable till day 8-9, then starts to decline. In this study 6-7 days infections were performed. HepaRG^{NTCP} cells were seeded at the density allowing cells to be sub-confluent the next day. After 4-5 days of culture the medium was supplemented with 1.8% DMSO and 1 µg/mL tetracycline. HDV infection was done on day 7-8 post seeding equally to HepaRG cell line. All infections were performed in duplicates or triplicates.

PHH cells were maintained in the medium supplemented with 2% DMSO and infected with HDV at MOI 30 as described above for HepaRG cells.

Drug treatment

Drug	Target	Concentration	Time
2-Deoxyglucose	Hexokinase inhibitor	5 mM	4 days
Sodium oxamate	Lactate dehydrogenase inhibitor	10 mM	4 days

CB839	Glutaminase inhibitor	10 µM	4 days
Albizziin	Asparagine synthetase inhibitor	10 mM	4 days
Azaserine	Purine biosynthesis inhibitor	10 µM	4 days
BSO	gamma-glutamylcysteine synthetase inhibitor	10 µM	4 days
Ruxolitinib	JAK inhibitor	1 µM	4 days
BX795	TBK1 inhibitor	3 µM	4 days
Torin 1	mTORC1 inhibitor	200 nM	24 hours
ISRIB	eIF2α inhibitor	1 µM	4 days
GSK2606414	PERK inhibitor	1 µM	4 days
C16	PKR inhibitor	1 µM	4 days
Triptolide	Transcription inhibitor	1 µM	kinetics
Rotenone	Complex I inhibitor	0.5 µM	2 days
Oligomycin A	Complex V inhibitor	0.25 µM	2 days
Sodium azide	Cytochrome oxidase inhibitor	0.25 mM	2 days
Doxorubicin	DNA topoisomerase II inhibitor	3 µg/mL	3 days

eIF2α inhibitors functional test

PERK inhibitor GSK2606414 was tested in the presence of PERK activator tunicamycin. HepaRG^{NTCP} cells were pre-treated with 1 µM GSK2606414 for 1 hour, then 2 µg/mL of tunicamycin was added for 4 hours. Control cells were non-treated or treated with only GSK2606414 or only tunicamycin.

PKR inhibitor C16 was tested in the presence of poly (I:C). HepaRG^{NTCP} cells were pre-treated with 1 µM C16 for 1 hour, then 5 µg/mL of poly (I:C) was transfected using lipofectamine 2000 in the presence or absence of C16 for 4 hours. Control cells were treated with only lipofectamine 2000 or C16 + lipofectamine 2000.

eIF2α inhibitor ISRIB was tested in the presence of tunicamycin similar to GSK2606414.

In all cases RNA was isolated and used for RT-qPCR analysis. CHOP mRNA level was used as a readout for eIF2α activation.

Stable shRNA cell lines

For the development of stable shRNA cell lines the lentiviral vector LeGO-G/Neo-opt was used. Each guide contains 19 nt target sequence; loop TTCAAGAGA; forward primer starts from T required for polymerase and ends with TTTTTTC (C is for XhoI restriction site); reverse primer starts with TCGA (for XhoI restriction site). Two guides targeting ATF4 and one control scrambled guide (listed in the corresponding table) were introduced into the vector using HpaI/XhoI restriction sites. The resulting plasmids were checked using PCR (primers pLEGO-seq), restriction analysis (HpaI site not retained) and forward/reverse sequencing (the loop did not allow to do a complete sequencing of the guide, so it has been done from both directions until the loop location). Each plasmid was transfected into HEK293T cell line along with 3 lentiviral packaging plasmids (pLP1, pLP2 and pVSV-G). $CaCl_2$/HBS transfection was used. For 50% confluent cells on 10cm dishes 4 µg of shRNA plasmid, 4 µg of pLP1, 3 µg of pLP2, and 0.8 µg of pVSV-G were added to 270 µL of water and mixed well. Then 28.8 µL of cold 2.5 M $CaCl_2$ solution were added slowly with simultaneous slow vortexing and mixed well. This mixture was slowly added to 300 µL of 2X HBS solution, then mixed well and incubated for 10 min at room temperature before adding it to the cell culture medium. 24h after the medium was changed. 48h post transfection the medium was collected, filtered through 0.45 µm filter and aliquoted. HepaRG^{NTCP} cells were transduced with 100 µL of the lentiviral stock per 1 mln cells and selected with 500 µg/mL of G418. The efficiency of the transduction was also controlled by GFP-positive cells. The efficiency of ATF4 shRNA guides was tested using ATF4 induction by tunicamycin treatment (western blotting) and by mRNA quantification by qPCR.

RNA extraction, DNAse treatment, reverse transcription

Total RNA was isolated using phenol-chloroform extraction (TRI reagent) with isopropanol-mediated RNA precipitation and the subsequent washes with 70% ethanol. The concentration and quality were assessed using Multiscan GO (Thermo Scientific) instrument. To exclude possible gDNA

contamination treatment with DNAse I was applied according to the manufacturer's protocol. 1 µg of DNA-free RNA was used for reverse transcription with random hexamers and M-MLV reverse transcriptase according to the manufacturer's protocol. The resulted cDNA was diluted 1:10 and used for qPCR.

SDS-PAGE/Western blot

Cells were washed 3 times with PBS and lysed in cold RIPA buffer containing protease and phosphatase inhibitors. The lysate was sonicated (Bioruptor Plus, Diagenode), and cleared by centrifugation at 12000g/10min/+4°C. Protein concentration was measured using BCA kit. 20 µg of total protein was used per lane (40 µg for ATF4 detection). Gel transfer on nitrocellulose membranes was performed using semi-dry Trans-Blot Turbo system (Bio-Rad) according to the manufacturer's protocol. The membranes were stained with Ponceau S solution, scanned and washed with TBS Tween 0.1% (TBST). The membranes were blocked with 5% non-fat milk or 3% bovine serum albumin (BSA) for phosphorylated proteins of interest. Blots were incubated with primary antibodies in 5% non-fat milk or 3% BSA overnight at +4°C, then washed with TBST and probed with secondary HRP-conjugated antibodies. After washing with TBST the blots were revealed using ECL or SuperSignal Femto substrates on GelDoc XR+ (Bio-Rad) instrument. β-actin was used for normalization. Densitometric quantification was performed using Image Lab 5.2.1 software.

Immunofluorescence

HepaRG^{NTCP} cells were seeded on cover slips and infected with HDV for 7 days as described above. Then cells were washed twice with PBS, fixed with 4% formaldehyde for 15 minutes, permeabilized with 0.1% Triton for 5 minutes, and blocked with 5% BSA in PBS at room temperature for 1 h. Cells were incubated with primary anti-HDAgs antibodies for 1 h at room temperature and subsequently labelled with secondary antibodies coupled to Alexa Fluor 488 (Thermo Scientific) for 1 h at room temperature. Cells were stained with 4′,6-diamidino-2-phenylindole (DAPI) for DNA staining and visualized using EVOS FL Auto Imaging System (Thermo Scientific).

Flow cytometry (DCFH2DA, MitoTracker green FM, MitoTracker Red CMXRos, MitoSOX)

HepaRG^{NTCP} cells were seeded in 24-well plates and infected with HDV for 7 days as described above. DCFH2DA staining: 20µM/30 min in DMEM without phenol red and FBS. MitoTracker green FM staining: 100 nM/10 min in complete medium. MitoTracker Red CMXRos staining: 300 nM/10 min in complete medium. MitoSOX staining: 7.5 µM/30 min in complete medium. Cells were washed once with PBS and harvested with trypsin. Trypsin was neutralized with complete medium, then cells were washed twice with DMEM without phenol red and FBS, resuspended in 300 µl of this medium and placed on ice. Immediately after that, the samples were run on the flow cytometer (BD FACSCalibur). Analysis was performed using CellQuest software.

Albumin ELISA

HepaRG^{NTCP} cells were infected with HDV for 7 days as described above. The cell culture supernatants were centrifuged at 3000g/10 min/+4°C to remove cells. The clarified supernatant was diluted 1:100 in the diluent solution provided with the kit. ELISA was performed according to the manufacturer's protocol. The absorbance at 450 nm was detected using Multiscan GO (Thermo Scientific) instrument.

Quantitative metabolite measurements and stable isotope tracing using liquid chromatography-coupled mass-spectrometry

HepaRG^{NTCP} cells were seeded in 6-well plates and infected with HDV for 7 days as described above. 5 replicates per condition were prepared. For steady state measurements the cells were grown under standard conditions, while for isotope enrichment analysis the cells were grown with uniformly labelled ^{13}C6-D-glucose (5.4 mM in growth media) or with uniformly labelled ^{13}C5-L-glutamine (1 mM in growth media). The cells were grown for the last 10 h or 3 h of the experiment, respectively. For the metabolite extraction the growth medium was removed followed by two washing steps with freshly prepared, pre-warmed (37°C), 75 mM ammonium carbonate solution (pH 7.4). The washed cells were then extracted with 400 µl of pre-cooled (-20°C) extraction buffer (methanol: acetonitrile: water (40:40:20 [v:v:v])) which was added to each well, and plates were incubated at -20°C for 10 min. Cells

were scraped and the resulted solution and the precipitated cellular material was transferred to pre-labelled 1.5 ml Eppendorf tubes, which were kept on ice. To collect all remaining cellular material, the sampling was repeated twice by addition of 400 µl of pre-cooled (-20°C) extraction buffer. The three pooled extracts (1.2 mL) were sonicated at +4°C (Bioruptor Plus, Diagenode) and centrifuged for 10 min at 4°C and 16000 g. The obtained precipitate was resuspended in 100 µl of RIPA buffer containing protease inhibitors and used for protein quantification by BCA kit. The cleared supernatant was transferred to a new tube and immediately dried down using a vacuum concentrator (Scan Speed 40, Labogene, Denmark). The dried metabolite pellets were stored at -80°C or used directly for liquid chromatography mass spectrometry analysis. Two wells without cells were treated equally and used as blank samples.

Anion-Exchange Chromatography High Resolution Mass Spectrometry (AEX-HRMS) for the analysis of anionic metabolites

The extracted and dried metabolites were resuspended in 150 µl of UPLC/MS grade water (Biosolve, Valkenswaard, Netherlands), of which 100 µl were transferred to polypropylene autosampler vials (Chromatography Accessories Trott, Germany) before AEX-MS analysis.

The samples were analysed using a Dionex ionchromatography system (Integrion Thermo Fisher Scientific) as described previously (40). In brief, 5 µL of the polar metabolite extract were injected in push partial mode, using an overfill factor of 1, onto a Dionex IonPac AS11-HC column (2 mm × 250 mm, 4 µm particle size, Thermo Fisher Scientific) equipped with a Dionex IonPac AG11-HC guard column (2 mm × 50 mm, 4 µm, Thermo Fisher Scientific). The column temperature was held at 30°C, while the auto sampler was set to 6°C. A potassium hydroxide gradient was generated using a potassium hydroxide cartridge (Eluent Generator, Thermo Scientific), which was supplied with deionized water (Milli-Q, IQ 700, Millipore). The metabolite separation was carried at a flow rate of 380 µL/min, applying the following gradient conditions: 0-3 min, 10 mM KOH; 3-12 min, 10−50 mM KOH; 12-19 min, 50-100 mM KOH; 19-22 min, 100 mM KOH, 22-23 min, 100-10 mM KOH 23-23.1. The column was re-equilibrated at 10 mM for 3 min.

Eluting compounds were detected in negative ion mode using full scan measurements in the mass range m/z 77–770 on a Q-Exactive HF high resolution MS (Thermo Fisher Scientific). The heated electrospray ionization (ESI) source settings of the mass spectrometer were: Spray voltage 3.2 kV, capillary temperature was set to 300°C, sheath gas flow 50 AU, aux gas flow 20 AU at a temperature of 330°C and a sweep gas glow of 2 AU. The S-lens was set to a value of 60.

Ultra Performance Liquid Chromatography-high-resolution mass spectrometry-based (UPLC-HRMS) analysis of amine-containing metabolites

The UPLC-HRMS analysis of amine-containing compounds was performed as described previously (41). In brief: the remaining 50 µL of the available 150 µL of the above mentioned (AEX-HRMS) polar phase were mixed with 25 µl of 100 mM sodium carbonate (Sigma), followed by the addition of 25 µl 2% [v/v] benzoylchloride (Sigma) in acetonitrile (UPC/MS-grade, Biosove, Valkenswaard, Netherlands). Derivatized samples were thoroughly mixed by pipetting and kept at RT (20°C) until analysis.

For the UPLC-HRMS analysis, 2 µl of the derivatized sample was injected onto a 100 x 2.1 mm HSS T3 UPLC column (Waters). The flow rate was set to 400 µL/min using a binary buffer system consisting of buffer A (10 mM ammonium formate (Sigma), 0.15% [v/v] formic acid (Sigma) in UPC-MS-grade water (Biosove, Valkenswaard, Netherlands)). Buffer B consisted of acetonitrile containing 0.1% formic acid (UPC-MS grade, Biosove, Valkenswaard, Netherlands). The column temperature was set to 40°C and the UPLC gradient was: 0-15% B 0 - 4.1 min; 15-17% B 4.1 – 4.5 min; 17 - 55% B 4.5-11 min; 55 - 70% B 11 – 11.5 min, 70-100% B 11.5 - 13 min; 100% B 13 - 14 min; 100-0% B 14 -14.1 min; 0% B 14.1-19 min. The connected mass spectrometer (Q-Exactive Plus, Thermo Fisher Scientific) was operating in positive ionization mode recording the mass range m/z 100-1000. The heated ESI source settings of the mass spectrometer were: Spray voltage 3.5 kV, capillary temperature 300°C, sheath gas flow 60 AU, aux gas flow 20 AU at 330°C and the sweep gas was set to 2 AU. The RF-lens was set to a value of 60.

Data analysis of the AEX-HRMS and UPLC-HRMS measurements

The semi-targeted raw data analysis of the AEX-HRMS and the UPLC-HRMS samples was performed using the TraceFinder software (Version 5.1, Thermo Fisher Scientific). The identity of each compound was validated by authentic reference compounds, which were measured at the beginning and the end of each sample set. For relative quantification of the unlabelled compound, the area of the deprotonated $[M\text{-}H^+]^{-1}$ or doubly deprotonated $[M\text{-}2H^+]^{-2}$ monoisotopic (M0) mass peak of every analysed compound was extracted and integrated using a mass accuracy <3 ppm and a retention time (RT) tolerance of <0.05 min as compared to the independently measured reference compounds.

The relative isotopic distribution of each compound was calculated from the proportion of the peak area of each isotopologue towards the sum of all isotopologues for the corresponding compound. No normalisation was applied as the number of quiescent differentiated HepaRG^{NTCP} cells does not change throughout the experiment. The later was confirmed by SRB method and also measuring labelled glucose and glutamine consumption. In addition to that the absence of difference in glucose consumption between mock and HDV-infected cells was confirmed measuring 2-NBDG (fluorescent glucose) uptake by flow cytometry.

The absolute ^{13}C-enrichment, namely the proportion of ^{13}C molecules traced in each compound, was calculated by multiplying the peak area of each detected isotopologue of a compound with the proportion of the ^{13}C and ^{12}C molecule number in the corresponding isotopologue. The obtained ^{13}C area fraction of each isotopologue are subsequently summed up, providing the ^{13}C enrichment value of each compound. Dividing this absolute ^{13}C area with the summed area of all isotopologues provides the relative ^{13}C enrichment factor.

Similarly to the semi-targeted data analysis for the AEX-HRMS samples, the analysis of the UPLC-HRMS measured Bz samples was performed. Here the peak area of $[M + nBz + H^+]^+$ ions, where nBz corresponds to the number of benzoyl groups (C_7H_4O) attached to the analysed compound, were extracted using a mass accuracy (<3 ppm) and a retention time tolerance of <0.05 min. Areas of the cellular pool sizes and the isotopic distribution and ^{13}C enrichments were calculated as described in the AEX-MS method.

RNAseq

HepaRG^{NTCP} cells were seeded in 6-well plates and infected with HDV for 7 days as described above. Cells were washed with PBS, harvested by scrapping and precipitated. The precipitates were stored in RNAlater solution at -80°C until further use. Total RNA was extracted from cell samples using Qiagen RNeasy Plus Mini kit following manufacturer's instructions (Qiagen, Hilden, Germany).

Library Preparation with Stranded Poly A selection and NovaSeq Sequencing

Extracted RNA samples were quantified using Qubit 2.0 Fluorometer (Life Technologies, Carlsbad, CA, USA) and RNA integrity was checked using Agilent TapeStation 4200 (Agilent Technologies, Palo Alto, CA, USA). Strand-specific RNA sequencing library was prepared by using NEBNext Ultra II Directional RNA Library Prep Kit for Illumina following manufacturer's instructions (NEB, Ipswich, MA, USA). Briefly, the enriched RNAs were fragmented for 8 minutes at 94 °C. First strand and second strand cDNA were subsequently synthesized. The second strand of cDNA was marked by incorporating dUTP during the synthesis. cDNA fragments were adenylated at 3'ends, and indexed adapter was ligated to cDNA fragments. Limited cycle PCR was used for library enrichment. The incorporated dUTP in second strand cDNA quenched the amplification of second strand, which helped to preserve the strand specificity. The sequencing library was validated on the Agilent TapeStation (Agilent Technologies, Palo Alto, CA, USA), and quantified by using Qubit 2.0 Fluorometer (ThermoFisher Scientific, Waltham, MA, USA) as well as by quantitative PCR (KAPA Biosystems, Wilmington, MA, USA). The sequencing libraries were multiplexed and clustered on the flowcell. After clustering, the flowcell was loaded on the Illumina NovaSeq 6000 instrument according to manufacturer's instructions. The samples were sequenced using a 2x150 Pair-End (PE) configuration Raw sequence data (.bcl files) generated from Illumina NovaSeq was converted into fastq files and de-multiplexed using Illumina bcl2fastq program version 2.20. One mismatch was allowed for index sequence identification.

Illumina single-end reads were checked up with FastQC 0.11.9, preprocessed with Trimmomatic 0.39 (adapter removal + trimming) and mapped to the reference human genome (GRCh38,

Ensembl release 104) using STAR 2.7.9a (42) with turned off novel splice junctions search. Next, using geneBody_Coverage tool (from RSeQC 4.0.1 toolkit) we ensured the equality of read coverage distributions (over the entire length of transcripts, from 5’ to 3’) between the analyzed samples (43). Read counts per gene were calculated with featureCounts tool from ‘subread’ 1.6.0 toolkit. Then, reads counts data were transferred to edgeR 3.36.0 R package (44), and differential expression analysis was made up using quasi-likelihood F-test. Gene Ontology, KEGG, Reactome, WikiPathways enrichment was done using topGO 2.46.0, clusterProfiler 4.2.2, ReactomePA 1.38.0 packages and GO.db 3.14.0 database. Additional visualization was made up with pathview 1.34.0 package and in-house scripts.
