# Supplementary material for: IFN-β production promotes metabolic rewiring and protection against oxidative stress in hepatitis delta virus-infected hepatocyte cultures
Source: Cell Death Dis. 2025 Jul 18;16(1):534. doi: 10.1038/s41419-025-07838-z (PMC12274286; doi:10.1038/s41419-025-07838-z)

Fig. 1A-1

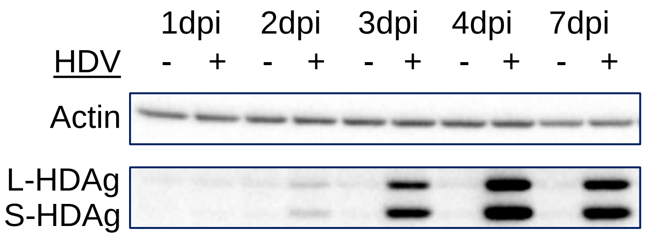

Exposure:

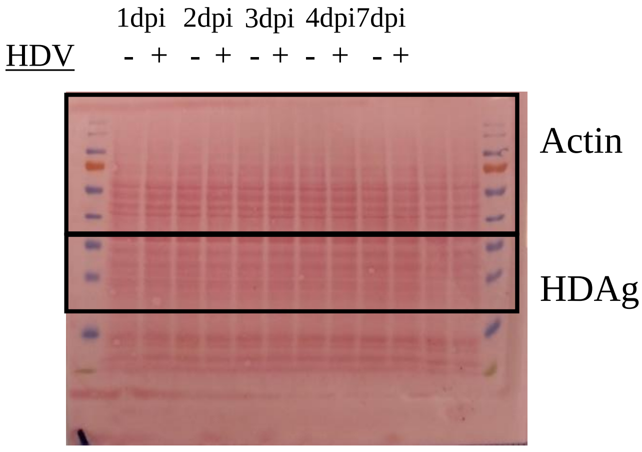

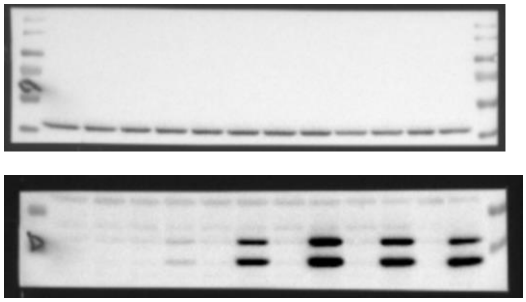

10 sec

1 sec

Ponceau S staining
Before membrane cutting

Merged image

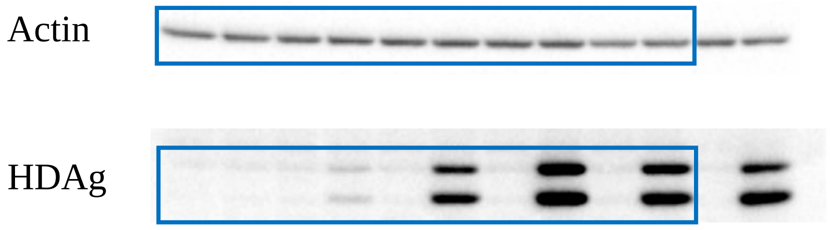

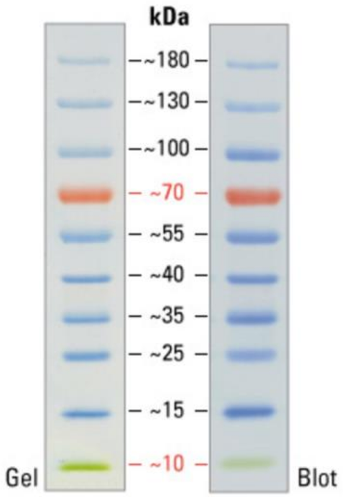

Fig. 1A-2

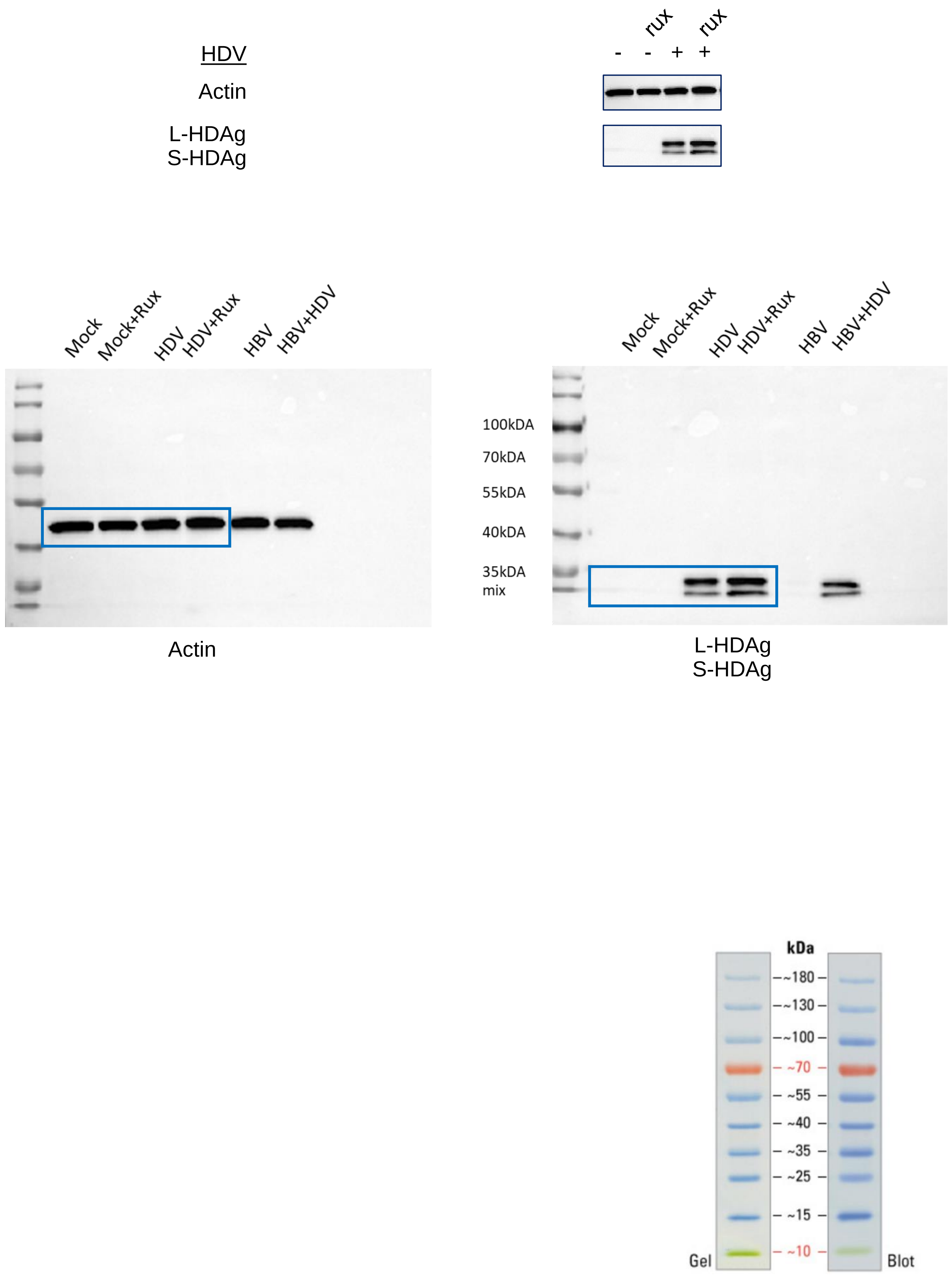

HDV
rux
rux
-
-
+
+
Actin
L-HDAg
S-HDAg
Mock
Mock+Rux
HDV
HDV+Rux
HBV
HBV+HDV
Actin
Mock
Mock+Rux
HDV
HDV+Rux
HBV
HBV+HDV
100kDA
70kDA
55kDA
40kDA
35kDA
mix
L-HDAg
S-HDAg
kDa
~180
~130
~100
~70
~55
~40
~35
~25
~15
~10
Gel
Blot

Fig. S1B

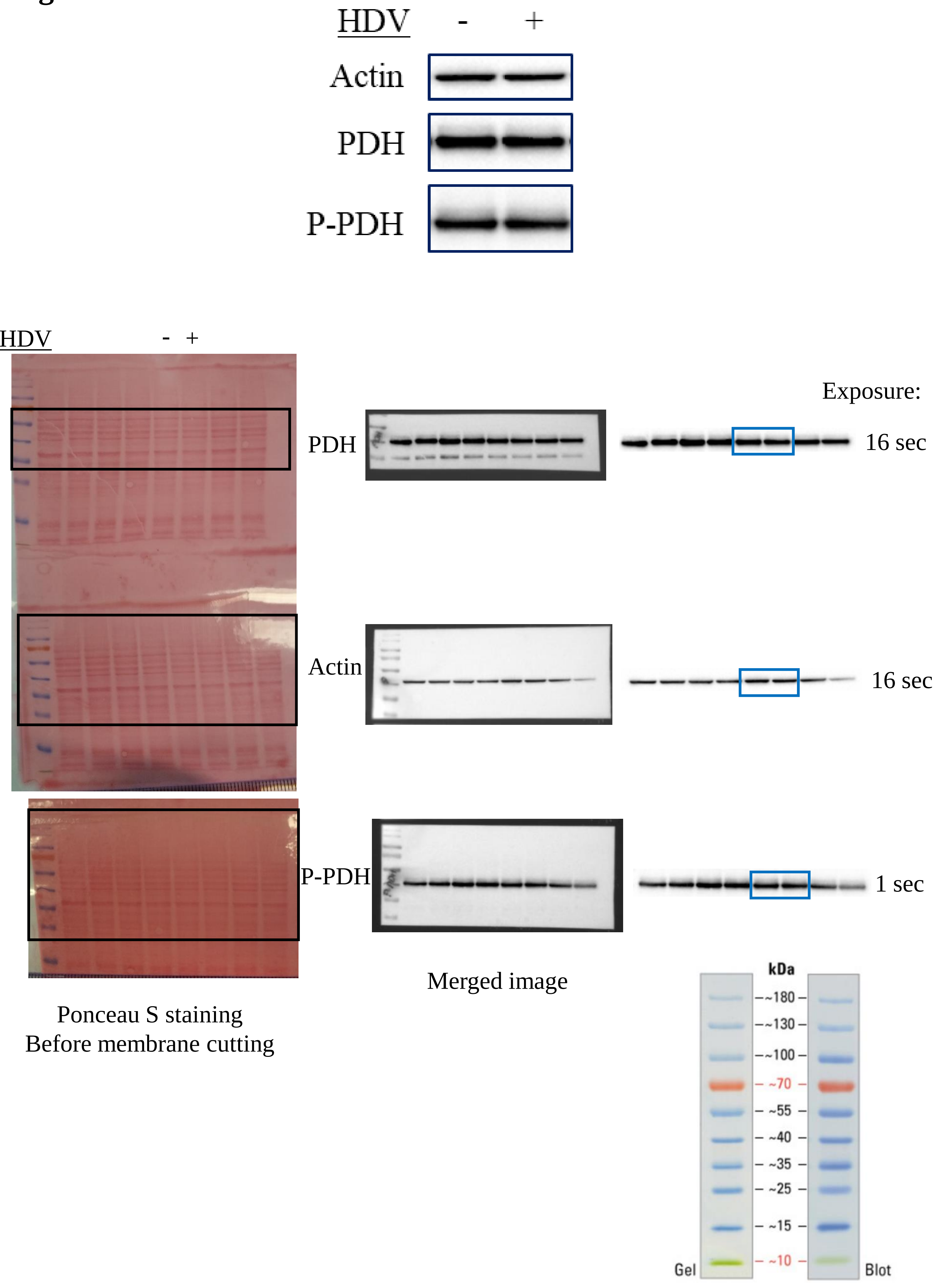
HDV
-
+
Actin
PDH
P-PDH
HDV
-
+
PDH
Actin
P-PDH
Exposure:
16 sec
16 sec
1 sec
Merged image
Ponceau S staining
Before membrane cutting
kDa
~180
~130
~100
~70
~55
~40
~35
~25
~15
~10
Gel
Blot

Fig. S1C

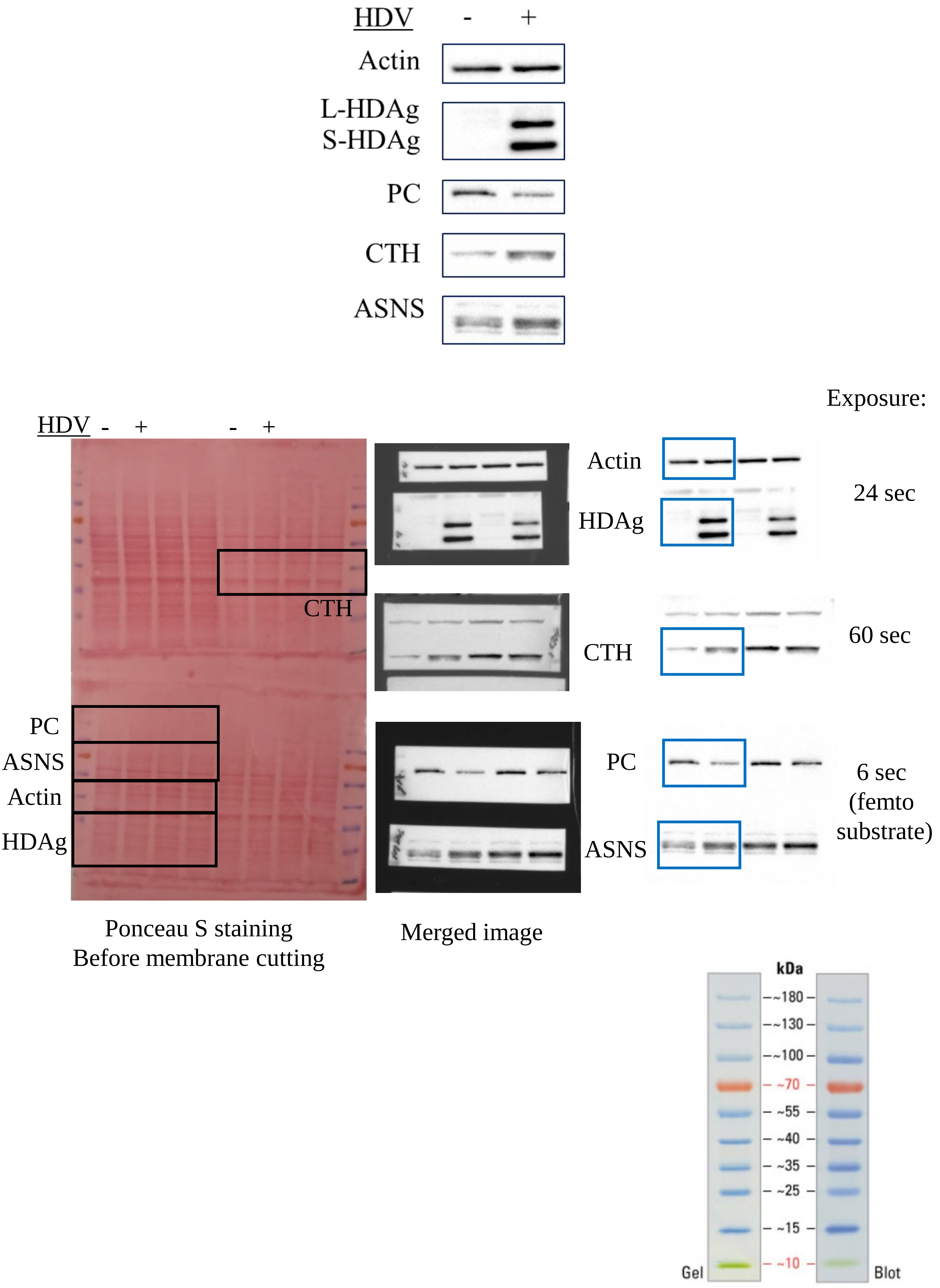

Ponceau S staining
Before membrane cutting

Merged image

Fig. S1C-2
(The same blot is also in Fig. 2A and Fig. 5C)

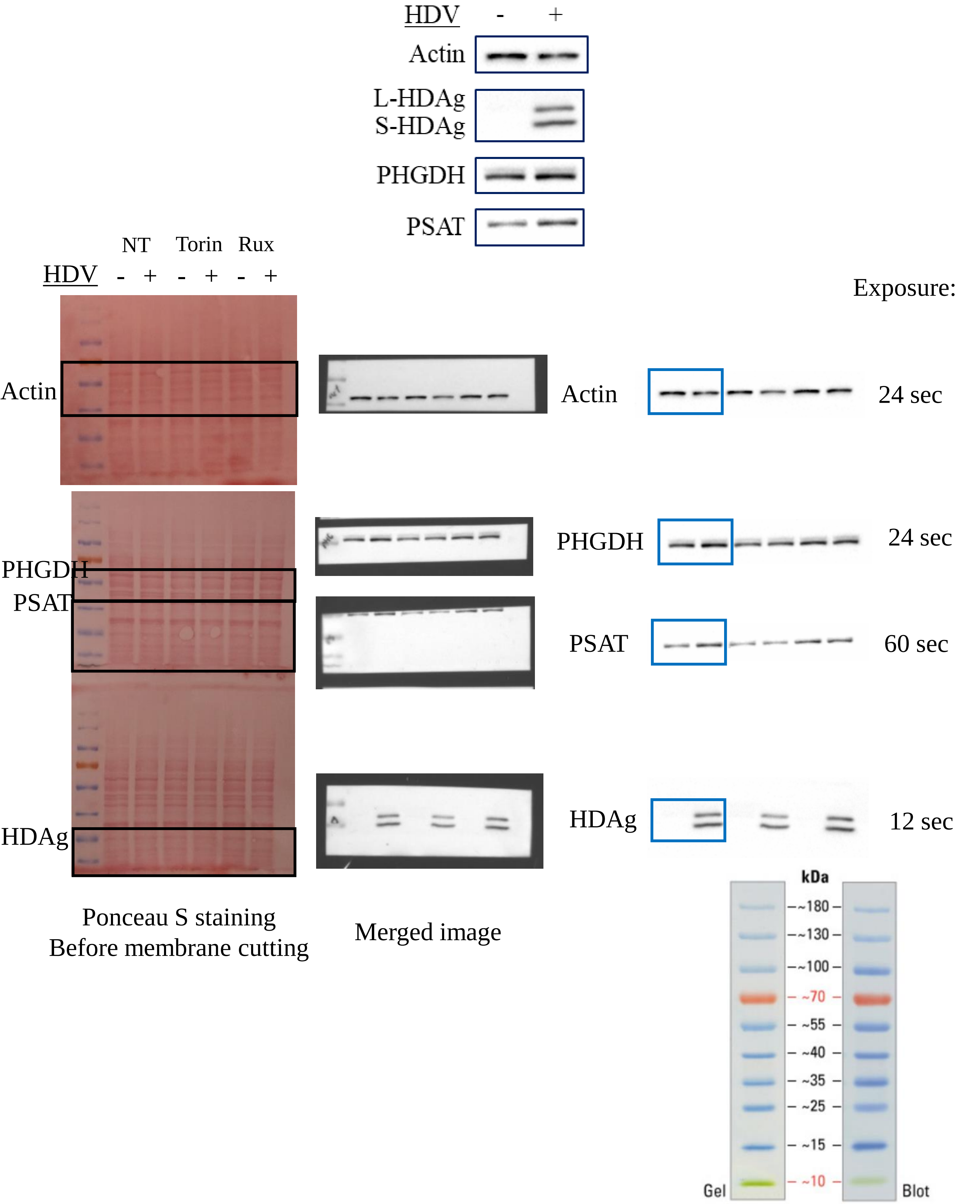

Fig. 2A
(The same blot is also in Fig. S1C and Fig. 5C)

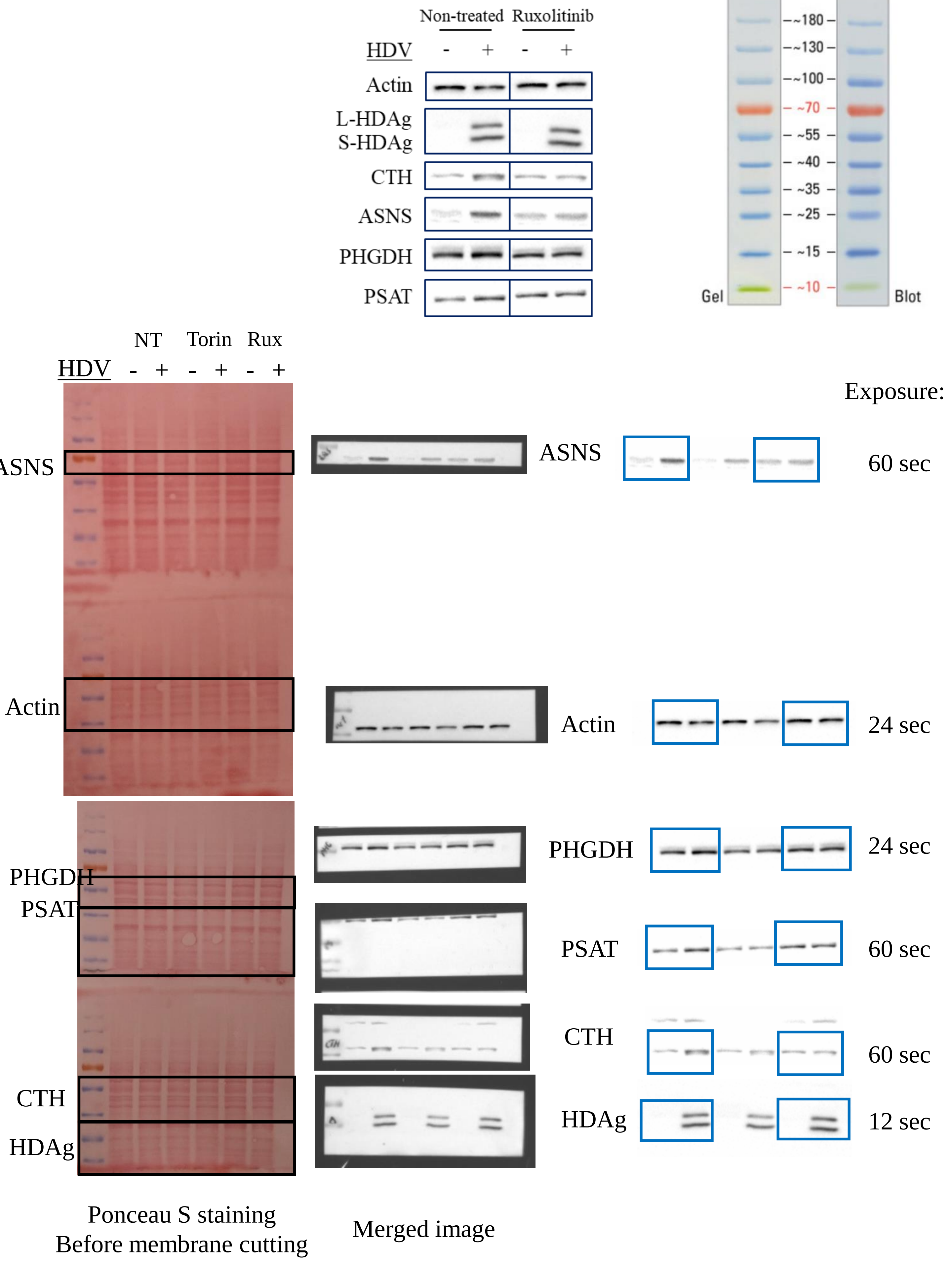

Fig. S2F

(The same blot is also in Fig. 5A)

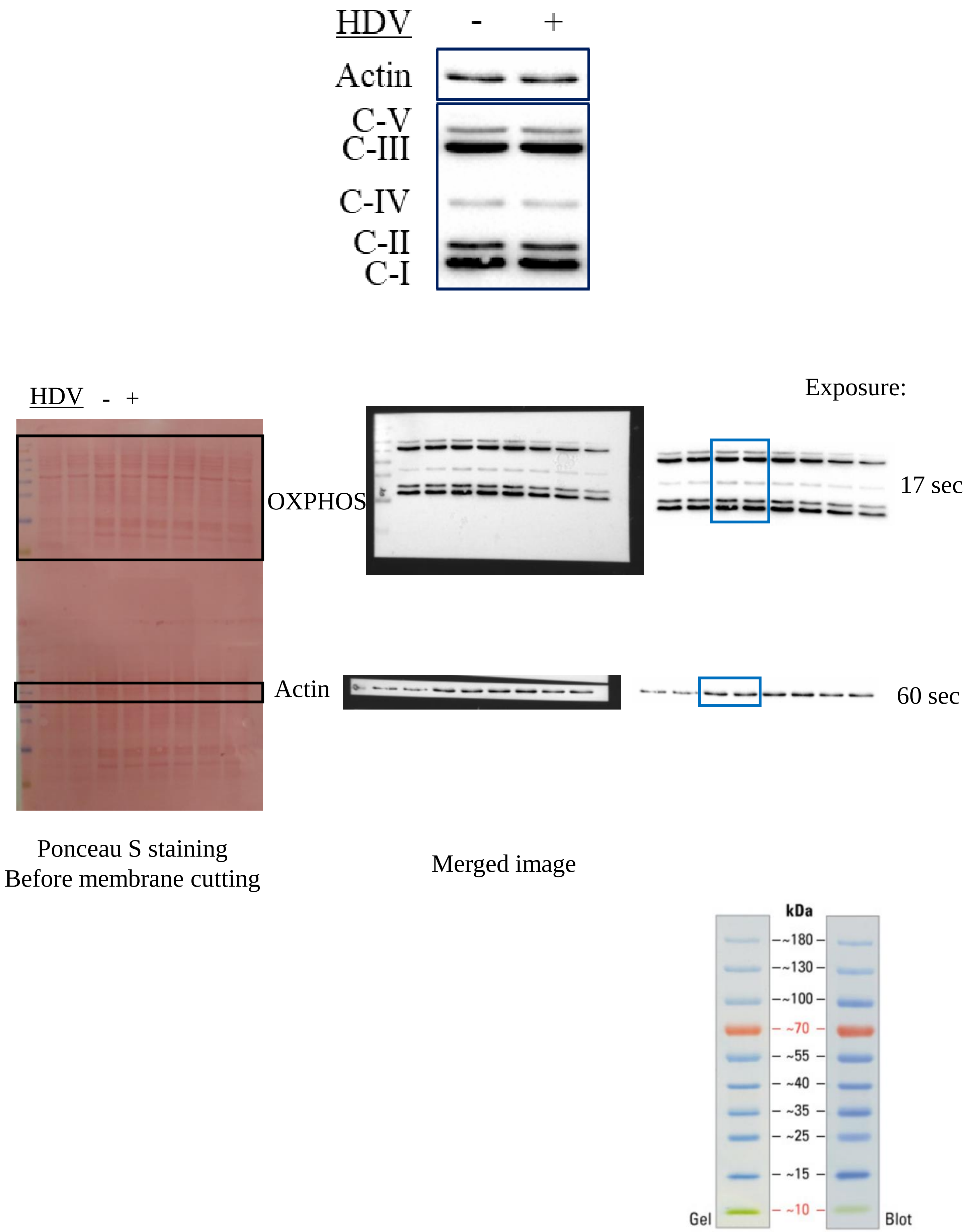

Fig. S2G

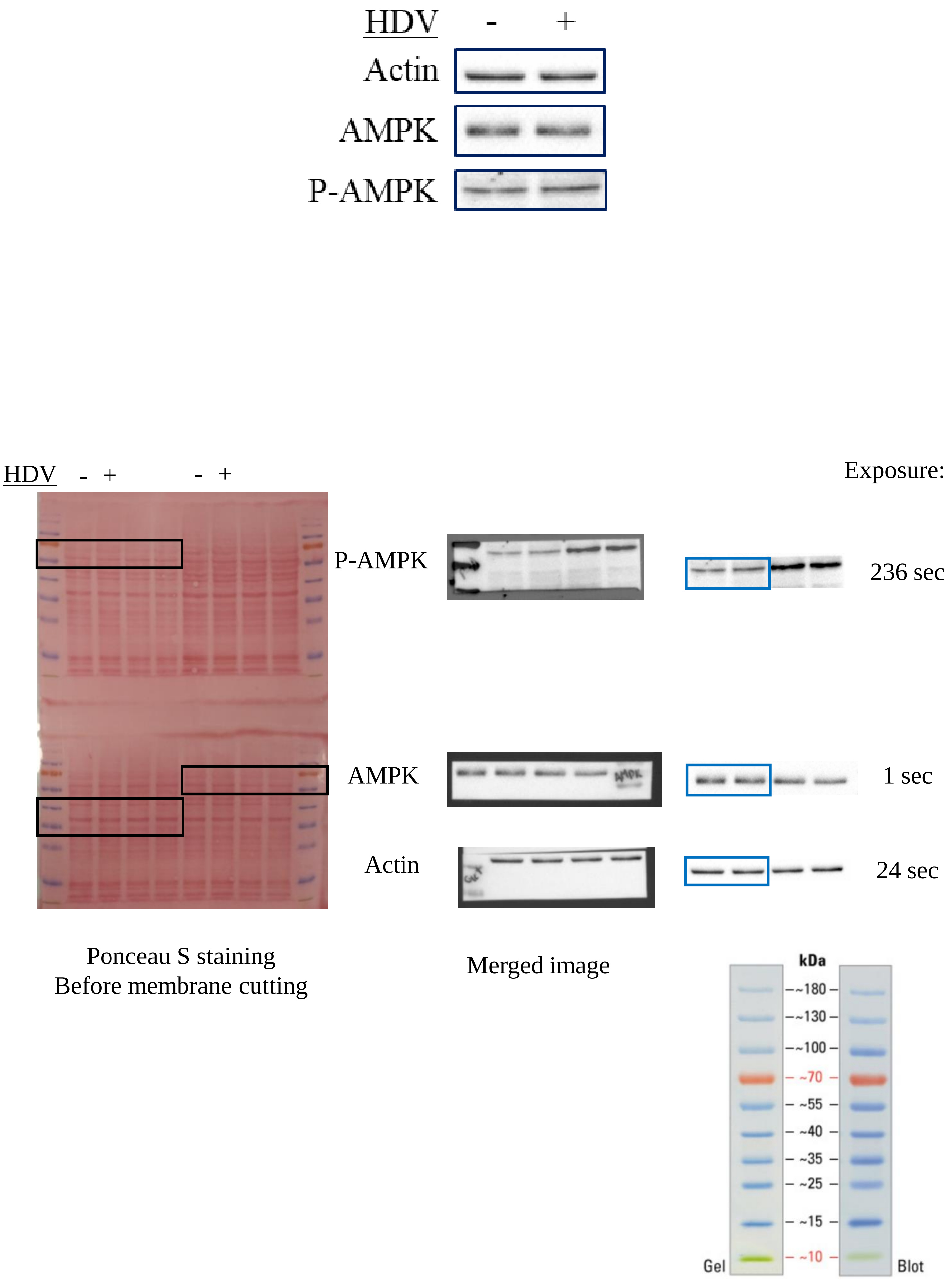
HDV - +
Actin
AMPK
P-AMPK
HDV - + - +
Exposure:
P-AMPK
236 sec
AMPK
1 sec
Actin
24 sec
Ponceau S staining
Before membrane cutting
Merged image
kDa
~180
~130
~100
~70
~55
~40
~35
~25
~15
~10
Gel
Blot

Fig. 5A
(The same blot is also in Fig. S2F)

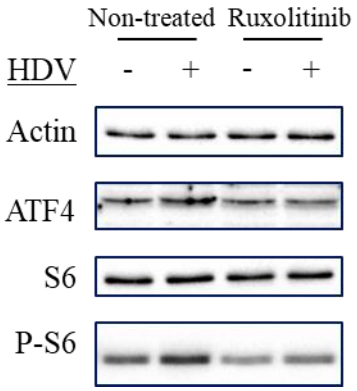

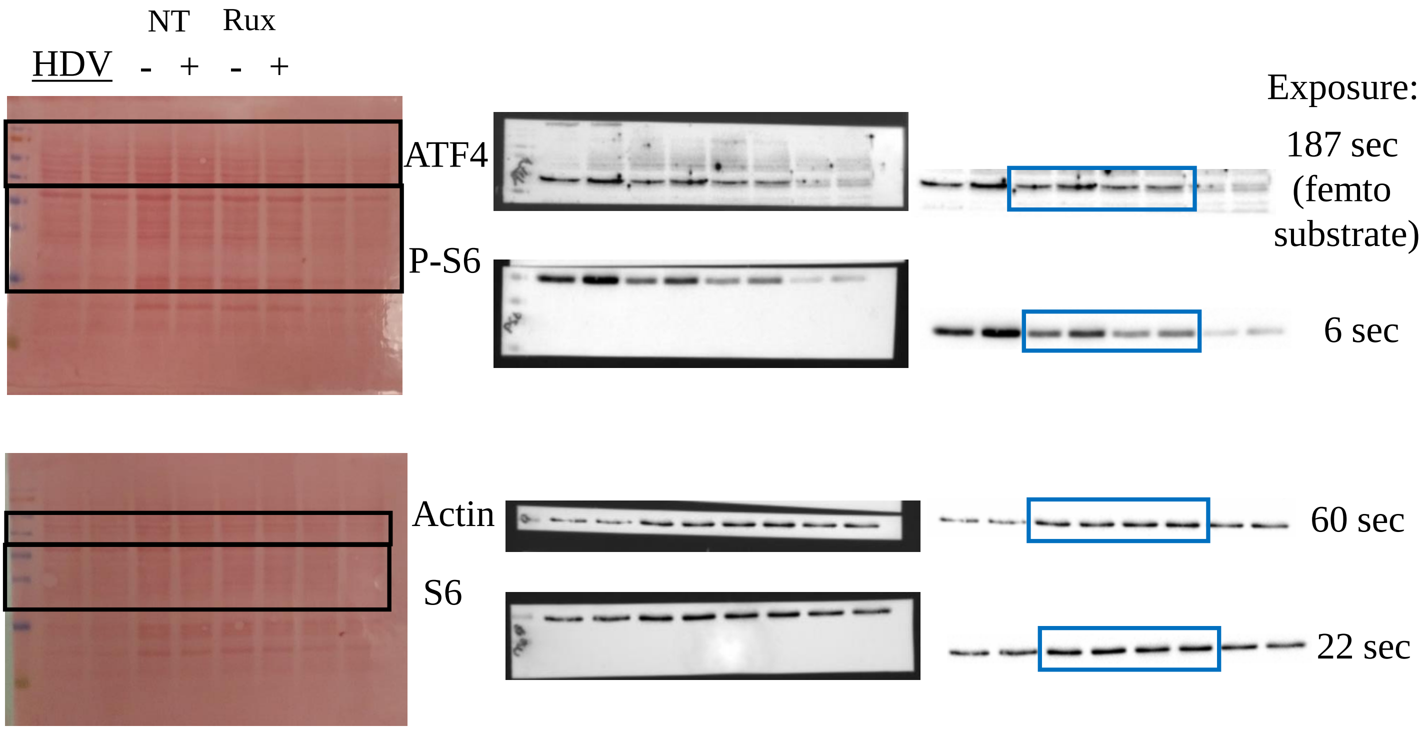

Ponceau S staining
Before membrane cutting

Merged image

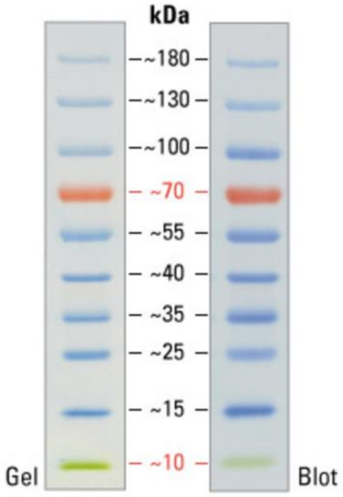

Fig. 5C
(The same blot is also in Fig. S1C and Fig. 2A)

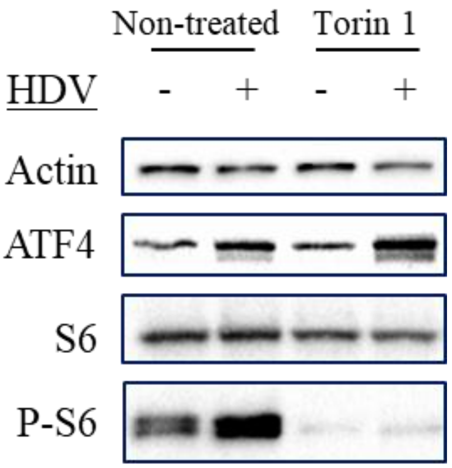

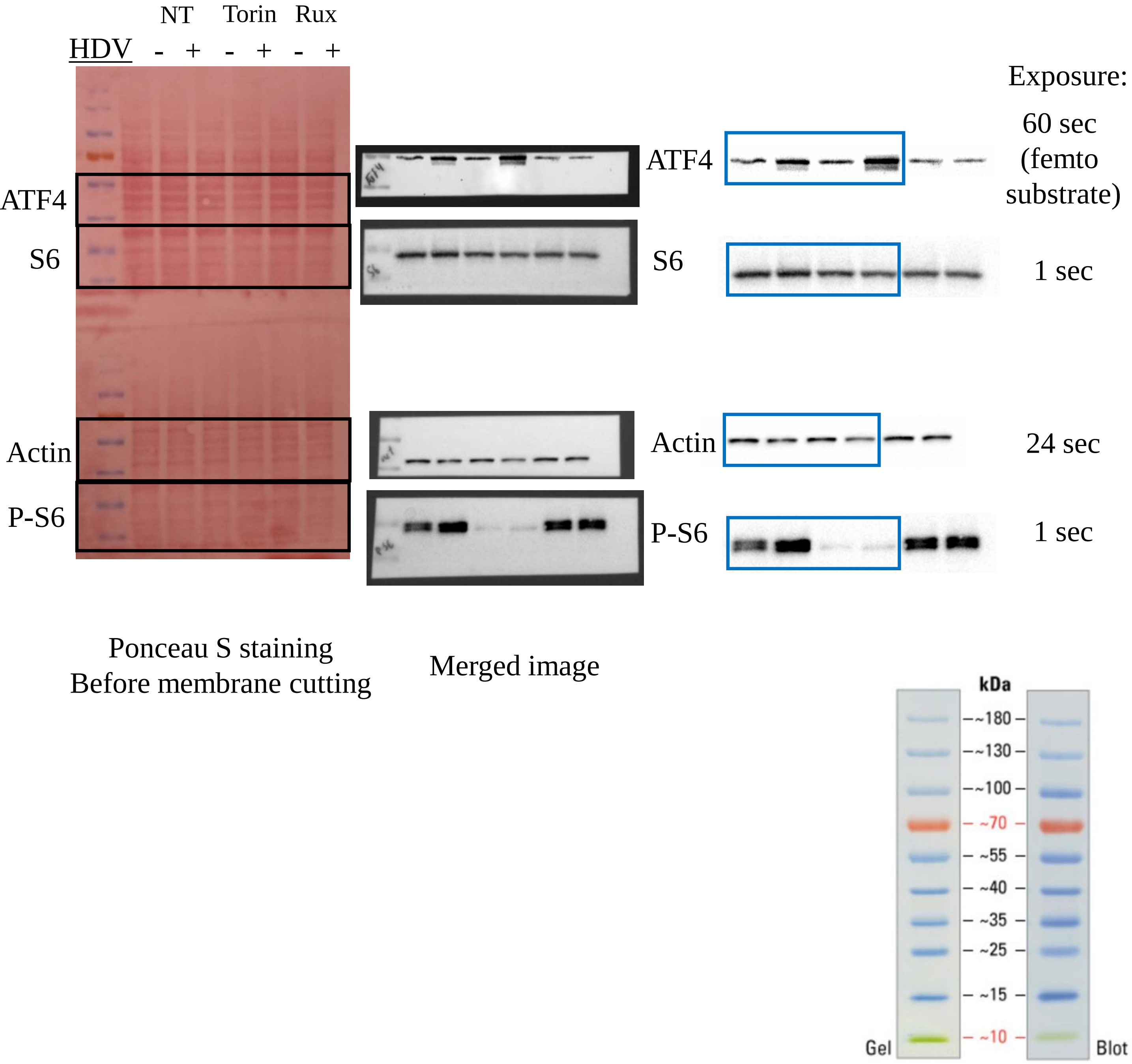

Ponceau S staining
Before membrane cutting

Merged image

Fig. S3A

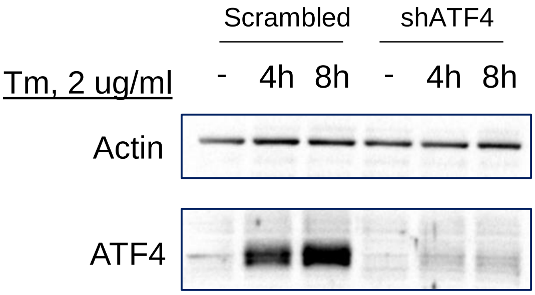

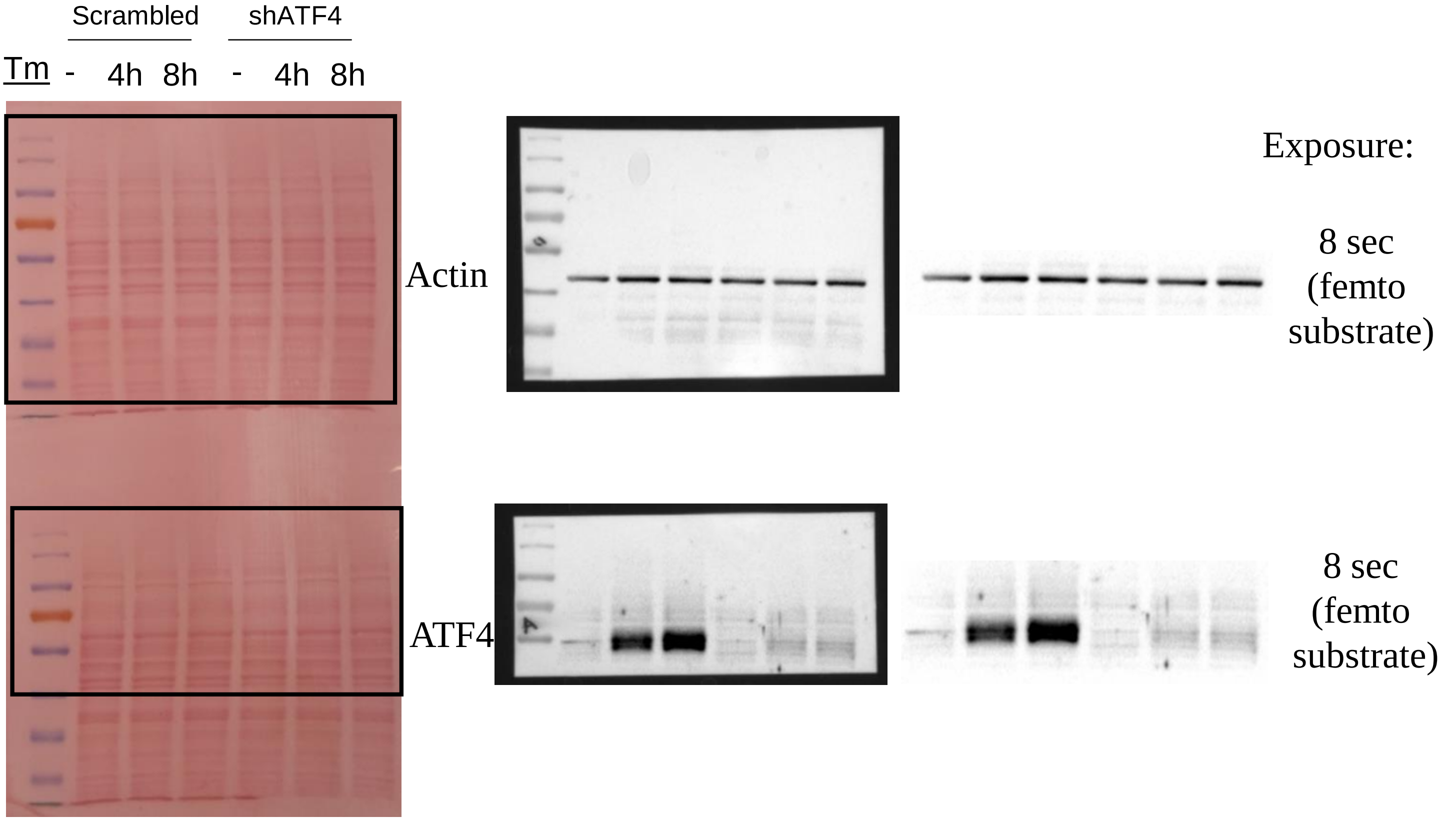

Ponceau S staining
Before membrane cutting

Merged image

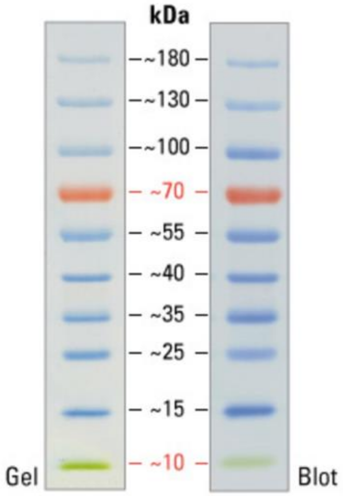

Supplement: Supplementary file 2 — Original immunoblots [file 41419_2025_7838_MOESM2_ESM.pdf]
